# Evolutionary Adaptation of the Fly Pygo PHD Finger toward Recognizing Histone H3 Tail Methylated at Arginine 2

**DOI:** 10.1016/j.str.2013.09.013

**Published:** 2013-12-03

**Authors:** Thomas C.R. Miller, Juliusz Mieszczanek, María José Sánchez-Barrena, Trevor J. Rutherford, Marc Fiedler, Mariann Bienz

**Affiliations:** 1 MRC Laboratory of Molecular Biology, Cambridge Biomedical Campus, Francis Crick Avenue, Cambridge CB2 0QH, UK

## Abstract

Pygo proteins promote Armadillo- and β-catenin-dependent transcription, by relieving Groucho-dependent repression of Wnt targets. Their PHD fingers bind histone H3 tail methylated at lysine 4, and to the HD1 domain of their Legless/BCL9 cofactors, linking Pygo to Armadillo/β-catenin. Intriguingly, fly Pygo orthologs exhibit a tryptophan > phenylalanine substitution in their histone pocket-divider which reduces their affinity for histones. Here, we use X-ray crystallography and NMR, to discover a conspicuous groove bordering this phenylalanine in the *Drosophila* PHD-HD1 complex—a semi-aromatic cage recognizing asymmetrically methylated arginine 2 (R2me2a), a chromatin mark of silenced genes. Our structural model of the ternary complex reveals a distinct mode of dimethylarginine recognition, involving a polar interaction between R2me2a and its groove, the structural integrity of which is crucial for normal tissue patterning. Notably, humanized fly Pygo derepresses Notch targets, implying an inherent Notch-related function of classical Pygo orthologs, disabled in fly Pygo, which thus appears dedicated to Wnt signaling.

## Highlights

• Coadapted mutations in fly Pygo PHD fingers alter their histone-binding surface
• A semi-aromatic groove in fly Pygo embeds dimethylated arginine 2 of histone H3
• Structural integrity of the arginine 2 groove is required for tissue patterning
• Humanized *Drosophila* Pygo derepresses Notch targets

## Graphical Abstract

**Figure:**
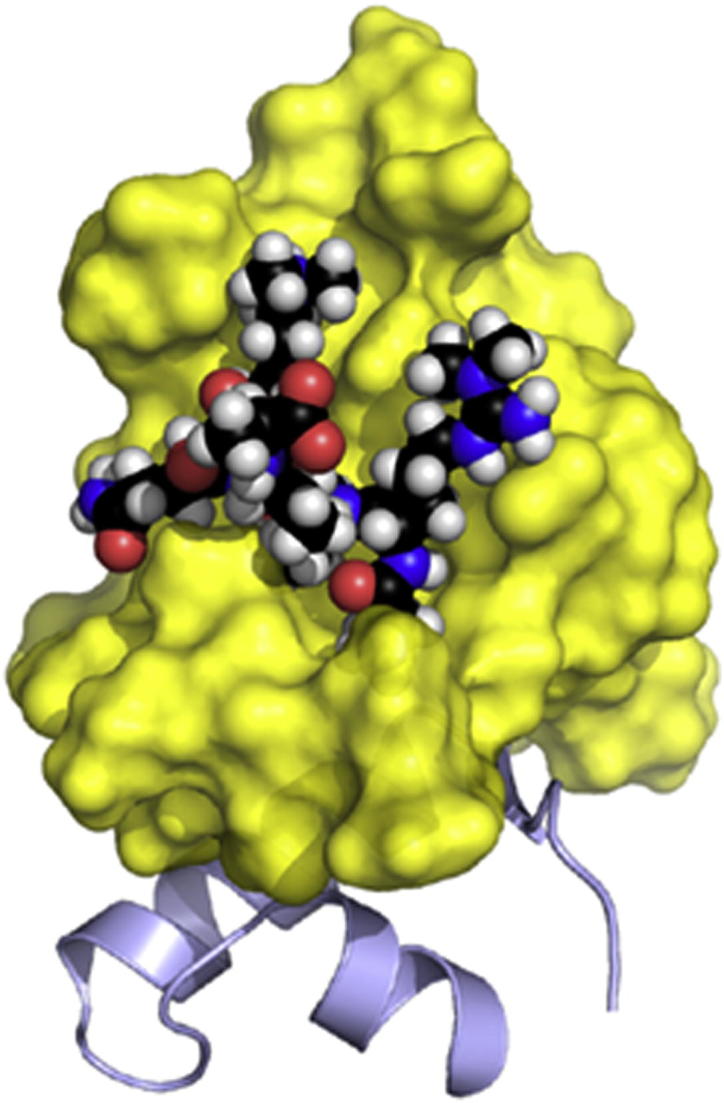

## Teaser

Miller et al. show that coadapted mutations in the *Drosophila* Pygo PHD finger alter its histone-binding surface to contain a novel semi-aromatic groove that embeds asymmetrically methylated arginine 2 of the histone H3 tail, a chromatin mark of silenced genes.

## Introduction

Wnt/β-catenin signaling controls numerous steps in the normal development and tissue homeostasis of animals (Cadigan and Nusse, 1997; Clevers, 2006). Hyperactivation of this pathway leads to many types of cancer, most notably colorectal cancer (Bienz and Clevers, 2000). Its key effector β-catenin (Armadillo in *Drosophila*) is stabilized in response to Wnt signaling, enabling it to bind to TCF/LEF DNA-binding proteins to activate Wnt-dependent transcription (Arce et al., 2006; Mosimann et al., 2009). Thus, the main read-out of this pathway is a transcriptional switch, which involves the recruitment of a range of transcriptional coactivators to the C terminus of β-catenin, including a SET methyltransferase that promotes the trimethylation of lysine 4 in the histone H3 tail (Sierra et al., 2006).

The transcriptional activity of Armadillo during *Drosophila* development depends on a highly conserved nuclear protein complex, consisting of Pygo (Pygo) and Legless (Lgs) (Belenkaya et al., 2002; Kramps et al., 2002; Parker et al., 2002; Thompson et al., 2002). Vertebrates encode two orthologs of each (Pygo1 and Pygo2, BCL9 and B9L/BCL9-2), which are required for efficient TCF-dependent transcription in Wnt-dependent tissue contexts (Gu et al., 2009; Li et al., 2007; Schwab et al., 2007; Song et al., 2007) and in human colorectal cancer cells with activated β-catenin (Adachi et al., 2004; Brembeck et al., 2004; de la Roche et al., 2008; Thompson et al., 2002). Lgs/BCL9 proteins are adaptors between Pygo and Armadillo/β-catenin, binding to Pygo PHD fingers through their homology domain 1 (HD1) and to Armadillo/β-catenin through their homology domain 2 (HD2; Kramps et al., 2002; Städeli and Basler, 2005). Rescue assays demonstrated that both interactions are essential for *Drosophila* development (Hoffmans and Basler, 2004; Townsley et al., 2004a).

Two models were proposed how Pygo and Lgs confer activity of Armadillo, both involving the Pygo > Lgs > Armadillo adaptor chain (Städeli and Basler, 2005). The first posits that Pygo is recruited through Lgs-Armadillo to *Drosophila* TCF (dTCF) target genes, i.e., exclusively during Wingless (Wg) signaling, to recruit an unknown transcriptional cofactor (Hoffmans et al., 2005; Kramps et al., 2002). The second envisages Pygo as an antirepressor, relieving Groucho-dependent repression at incipient Wg signaling (Mieszczanek et al., 2008), facilitating recruitment of Armadillo to dTCF target genes via Lgs (Townsley et al., 2004b). Accordingly, Pygo is associated with dTCF targets regardless of Armadillo and Wg (de la Roche and Bienz, 2007).

Support for the second model came from the discovery that the mammalian Pygo PHD fingers bind to methylated histone H3 tail (Fiedler et al., 2008; Gu et al., 2009; Miller et al., 2010). Interestingly, the histone-binding affinities of human Pygo PHD fingers are enhanced by their binding to HD1, ascribing a cofactor role to BCL9/B9L in promoting Pygo’s histone binding. The underlying mechanism is an allosteric communication, triggered by HD1 binding to PHD and relayed to its histone-binding surface through the PHD core (Miller et al., 2010).

**Figure 1:**
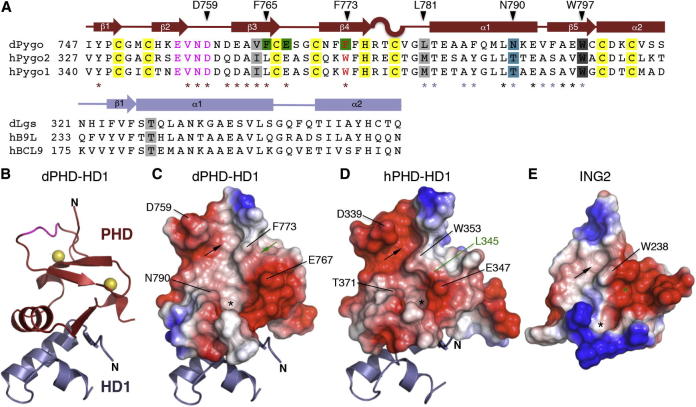
Structure of the *Drosophila* PHD-HD1 Complex (A) Alignments of PHD and HD1 sequences of *Drosophila* and human proteins; above, key residues and secondary structure elements (β, β strand; α, α helix; S, α turn); all *Drosophila* residues shown are visible in the crystal structure. Asterisks indicate residues involved in binding to H3K4me (maroon) or HD1 (blue-gray), or both (black, A1 pocket); red, pocket-divider; magenta, EVND motif; green, R2 groove; turquoise, T3 channel; gray, allosteric triplet (including PHD signature residue, dark gray); yellow, Zn^2+^-coordinating residues. (B) Ribbon representation of structure of dPHD-HD1 complex, colored as in (A). (C–E) Molecular surface representations of PHD structures, as indicated, colored according to electrostatic potential (red, negative; blue, positive), in complex with HD1 (ribbon representations in (C) and (D); (C) same view as in (B); histone-binding pockets are indicated by black arrows (K4me) or asterisks (A1), or in green (arrow, R2 groove; asterisk, R2 pocket in ING2, occupied by L345 in hPygo2); key residues are labeled. See also Figure S1.

Pygo orthologs belong to the subclass of PHD proteins whose recognition of histone H3 strictly depends on its methylation at lysine 4 (H3K4me), a chromatin mark associated with actively transcribed genes (Musselman and Kutateladze, 2011; Taverna et al., 2007). Most PHD fingers of this subclass exhibit three pockets connected by a narrow channel embracing threonine 3 of histone H3 (T3 channel; Figure 1): the N-terminal alanine (A1) is buried in a deep anchoring pocket, adjacent to a shallower pocket binding arginine 2 (R2), which is separated by a conserved tryptophan (W, the “pocket-divider”) from a deep aromatic pocket embedding K4me. Notably, some of these PHD fingers (e.g., those of SET-1 and TAF3; Kirmizis et al., 2007; Vermeulen et al., 2007) are highly sensitive to R2 methylation, and thus cannot bind to asymmetrically dimethylated R2 (R2me2a), a mark associated with silenced loci (Guccione et al., 2007; Kirmizis et al., 2007). This is because the terminal (unmodified) guanidinium group of the R2 side chain is buried in classical R2 pockets (e.g., Peña et al., 2006), explaining why its methylation can block binding to its pocket. However, human Pygo proteins are different: their R2 cavity is filled by the bulky side-chain of a leucine (L), which redirects the R2 side chain into the solvent, thus exposing its guanidinium group (Fiedler et al., 2008; Miller et al., 2010). Therefore, human Pygo is insensitive to R2 methylation, binding equally well to unmodified and methylated R2. This applies probably to most Pygo orthologs because this pivotal L is conserved in all vertebrates, and in most invertebrates all the way to sponges (Figure S1 available online). However, it is substituted by a phenylalanine (F) in some flies, with interesting structural and functional consequences (see Results).

Another striking substitution occurred in the brachycera suborder of the arthropods, including all Drosophilids, and also in a nematode Pygo ortholog (Figure S1), whose pocket dividers are F instead of W. Indeed, a recent study concluded that the sole function of the *Drosophila* Pygo PHD finger is its Lgs binding, while its histone binding is dispensable for development (Kessler et al., 2009). This contrasted our own conclusion, also based on *pygo* rescue assays, that the histone binding of fly Pygo, despite being weak, is functionally relevant (Fiedler et al., 2008). This issue of chromatin binding by *Drosophila* Pygo therefore remains controversial.

To resolve this, and to investigate how the deviant pocket-divider of the Pygo PHD finger affects histone binding, we solved the crystal structure of the *Drosophila* PHD-HD1 complex and examined its H3K4me binding by nuclear magnetic resonance (NMR) spectroscopy. This revealed a conspicuous semi-aromatic groove bordered by the pocket-divider (F773). We present a structural model, based on integrating intermolecular nuclear Overhauser effects (NOEs) with our crystal structures, that this groove embeds R2me2a whose cation undergoes a tight polar interaction with E767 (the groove wall opposite F773), representing a distinct mode of dimethylarginine recognition, different from that of Tudor domains (Tripsianes et al., 2011). Rescue assays in flies demonstrate that the structural integrity of the R2 groove and T3 channel is crucial for normal tissue patterning. Interestingly, a Pygo gain-of-function (gof) mutant with a humanized pocket-divider (F > W) has increased histone binding affinity, and acquires derepressive activity toward Notch targets. Like Wg targets, these are subject to Groucho-dependent repression (Jennings and Ish-Horowicz, 2008). A corollary is that classical Pygo orthologs have an inherent ability to derepress Notch-dependent transcription, which is disabled in fly Pygo by the W > F substitution in its deviant PHD finger.

## Results

We coexpressed Pygo PHD with Lgs HD1 in bacteria and purified the complex (dPHD-HD1) essentially as described (Fiedler et al., 2008). Although dPHD-HD1 is far less stable than the human complexes, we obtained diffracting crystals under several conditions, which allowed us to solve its structure at 2.7 Å resolution (Table 1; Figure S2). Overall, this is similar to that of its human counterparts, with an rmsd of 0.85 Å relative to 2vpb (Fiedler et al., 2008). Like the latter, dPHD binds two Zn^2+^ ions in a cross-braced fashion through two pairs of anti-parallel β strands (β1–4), and to HD1 through its single α helix (α1) and adjacent β strand (β5; Figures 1A–1C; see also below).

### A Striking Groove in the Histone-Binding Surface of *Drosophila* Pygo PHD

Opposite the PHD-HD1 interface are two conspicuous pockets, predicted to accommodate K4me and A1 (Fiedler et al., 2008; Miller et al., 2010; Figures 1A–1C): the semi-aromatic K4 pocket is bordered by an aspartate (D759, from the Pygo-defining EVND signature motif; Bienz, 2006) and by F773 (substituting for the W pocket-divider, as mentioned above), and is connected to the A1 anchoring pocket by a relatively straight channel for T3 (Figure 1C).

An important structural consequence of the W > F substitution is a significant widening of the T3 channel: this is bordered on one side by F773 (Figure 1C) the phenyl ring of which is less protruberant than the indole ring of a typical W pocket-divider (Figure 1D). This is partially compensated for by a T > N substitution at the opposite channel wall where the bulky side chain of N790 protrudes into the unliganded channel and thus narrows it (Figure 1C). In classical Pygo orthologs, the same position is occupied by a conserved T (Figure 1D; Figure S1) whose shorter side chain protrudes less into the unliganded channel (Fiedler et al., 2008; Miller et al., 2010).

**Figure 2:**
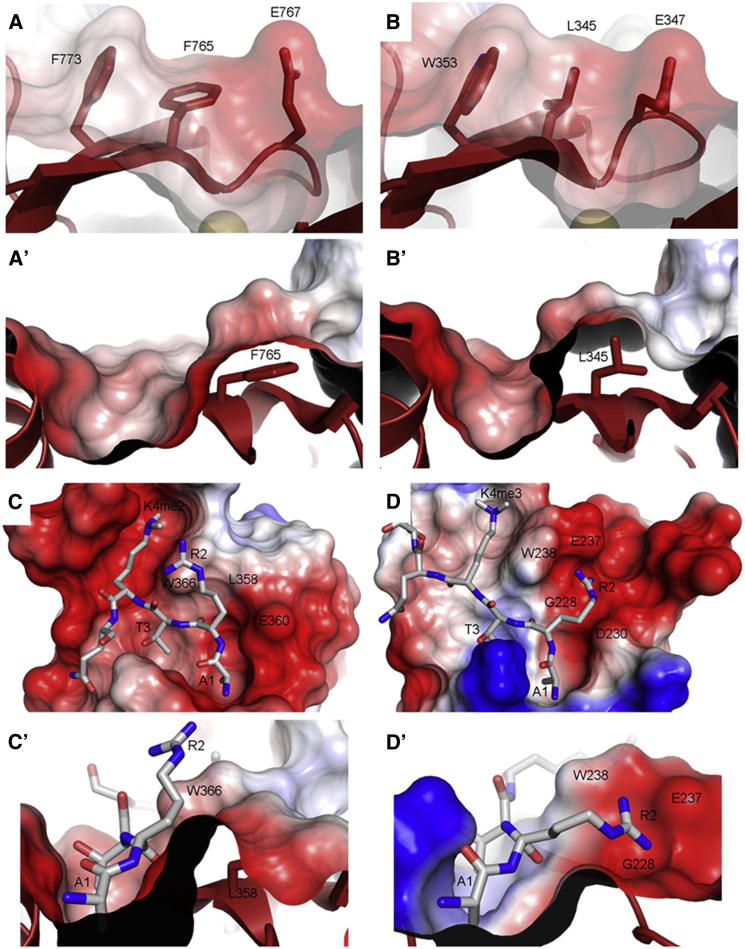
The R2 Groove in *Drosophila* Pygo (A and B) Close-up views of (A) R2 groove of fly Pygo and corresponding region of (B) hPygo2 (in surface and ribbon representations; coloring as in Figure 1); (A′ and B′) sagittal sections; floor and wall residues in stick representations. (C and D) Close-up views of R2 pockets in (C) hPygo2 or (D) ING2 (as in Figure 1; sagittal sections in C′ and D′), with bound H3K4me (stick representations; red, oxygen; blue, nitrogen), showing R2 guanidinium groups (C′) solvent-exposed or (D′) buried in pocket.

Abutting the F pocket-divider in fly Pygo is a striking groove, walled by F773 and E767, and floored by the aromatic ring of F765 (Figures 1C and 2A). This groove is not seen in human Pygo in which a conserved L (L345) fills out the equivalent space, substituting for F765 (Figures 1D, 2B, and 2C), as already mentioned. In classical PHD fingers (e.g., from ING2), a deep cavity is found in this position which buries R2 (Figures 1E and 2D). Therefore, *Drosophila* Pygo PHD evolved a distinct feature—the R2 groove.

### The R2 Groove Interacts with R2me2a

We attempted to determine the crystal structure of the ternary complex with H3K4me peptides, using previously successful strategies (Fiedler et al., 2008), e.g., linking the two domains by a flexible linker (dPHD-HD1link), which proved crucial for solving the structure of the human paralog complex (Miller et al., 2010). None of this was successful, possibly because of the low histone-binding affinity of the fly complex (see below).

**Figure 3:**
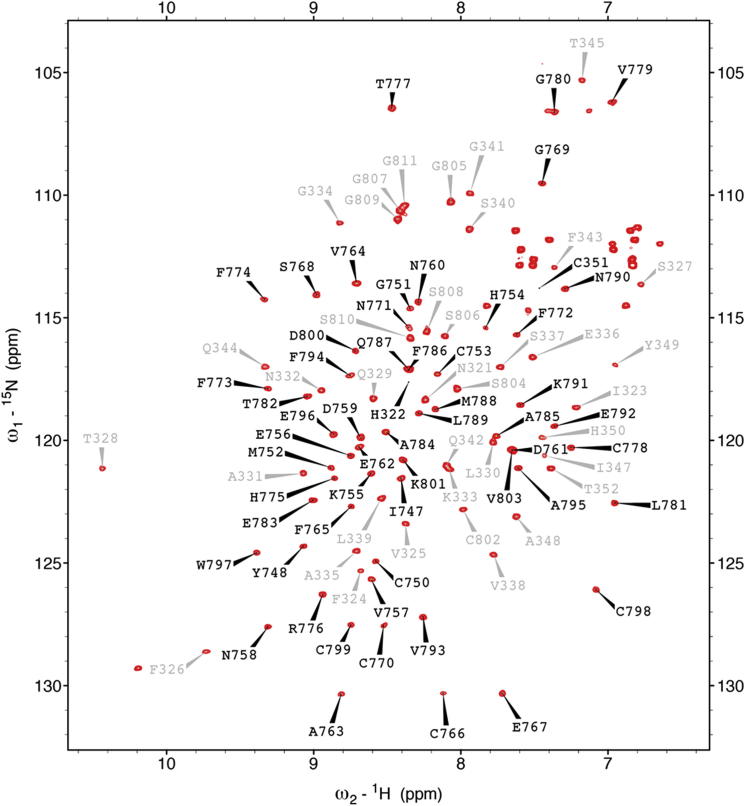
Assignments of PHD-HD1 Residues HSQC spectrum of 400 μM ^13^C-^15^N-labeled PHD-HD1link, with individual assigned residues annotated (PHD, black; HD1, gray).

**Figure 4:**
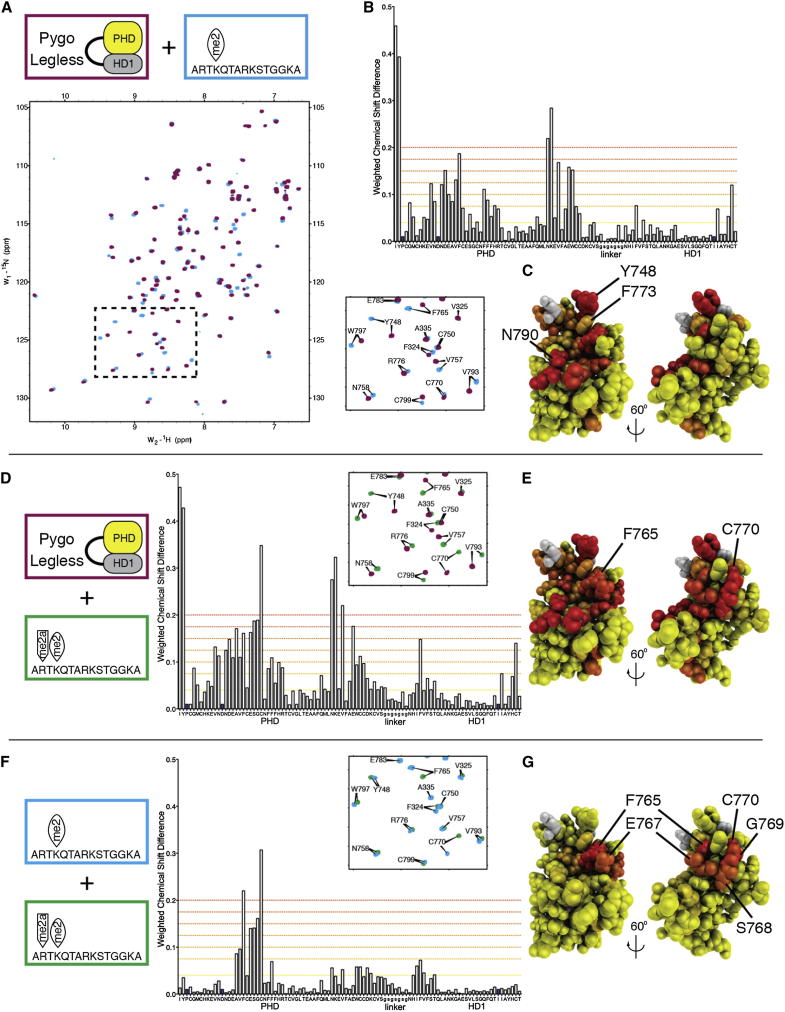
Interaction of the R2 Groove with Dually Modified Histone H3 Tail (A) Overlay of HSQC spectra of 50 μM ^15^N-labeled PHD-HD1link + 1 mM H3K4me2 (cyan) onto PHD-HD1link alone (magenta); inset, zoomed view of boxed area containing key residues (including F765 and C770). (B) Chemical shift difference map, showing backbone N-H CSP differences between PHD-HD1link ± H3K4me2, as calculated from the HSQC spectra in (A), plotted against the primary sequence (small letters indicate linker; blue bars, unassigned residues); weighted chemical shift differences represent absolute values of (change in ^1^H shift) + (change in ^15^N shift /5; Hajduk et al., 1997); dashed lines indicate increasing levels of CSPs, ranging from weak (yellow) to strong (red). (C) Heat maps of CSPs projected onto PHD-HD1 (structure on the right rotated by 60°, for side view of R2 groove), color-coded as in (B). (D and E) Map as in (B), and zoomed area of spectral overlay of 50 μM ^15^N-labeled PHD-HD1link + 1 mM H3R2me2aK4me2 (green) onto PHD-HD1link alone (magenta), and (E) corresponding heat maps, with color-coding as in (A)–(C). (F and G) Differential CSP map and zoomed area of spectral overlays from (A) and( D), and (G) corresponding heat maps, revealing residues that are differentially affected by dually- versus singly-modified histone H3 peptide; color-coding as in (A)–(C). See also Figures S3 and S4.

We therefore turned to NMR as a highly sensitive probe for intermolecular interactions in solution. We purified ^13^C-^15^N-labeled dPHD-HD1link, for NMR backbone resonance assignments (Figure 3). We then recorded heteronuclear single-quantum correlation (HSQC) spectra of ^15^N-labeled dPHD-HD1link, with or without our standard H3K4me2 15-mer (Fiedler et al., 2008). We thus observed clear chemical shift perturbations (CSPs; Figures 4A and 4B). Mapping these onto the crystal structure identified several residues in the predicted histone-binding surface (Figure 4C), most notably Y748 (K4 pocket lid), N790, and F765 (see below), suggesting that these residues interact directly with H3K4me2 (although CSPs can also reflect “bystander” effects, e.g., on sequence neighbors such as I747 and K791, neither of which contribute directly to the histone-pocket lining). HSQC-based titrations indicate an affinity of ∼1 mM to histone H3 peptides (Figure S3), considerably lower than previously thought (Fiedler et al., 2008).

Interestingly, a dually modified histone H3 peptide (H3R2me2aK4me2) induces generally more pronounced CSPs than singly-modified peptide (Figure 4D; Figure S3): in addition to the previously described CSPs, we also observe a strong CSP of C770 (Figure 4E), which supports the R2 groove floor. Intriguingly, the CSP of F765 (the R2 groove floor residue) is qualitatively different from that induced by H3K4me2, indicating a distinct interaction with the dually modified peptide. As expected, histone H3 tail binding to PHD-HD1 depends on K4 methylation because neither unmodified peptide nor H3R2me2a induces significant CSPs (Figure S4).

To pinpoint the residues specifically affected by R2me2a, we generated a differential CSP map (comparing dually versus singly modified peptides; Figure 4F); this identified C770 and F765 as the top differentially affected residues, but also E767 (R2 groove wall), S768 and G769 (supporting the R2 groove floor), and V764 (an allosteric triplet residue, see below)—altogether clearly highlighting the R2 groove (Figure 4G), indicating that R2me2a interacts specifically with this groove.

### A Distinct Mode of Dimethylarginine Recognition

**Figure 5:**
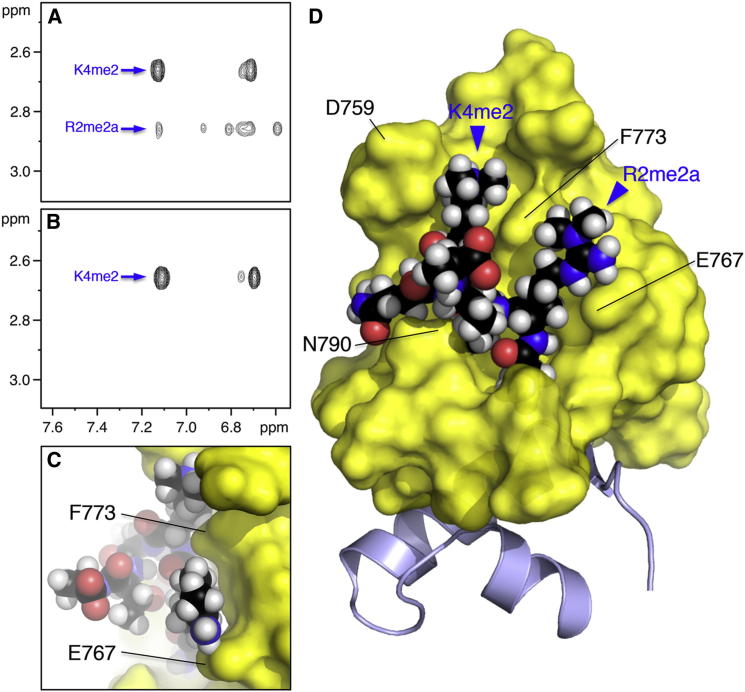
Interaction of R2me2a with Its Cognate Groove (A and B) Expanded views of *N*-methyl to aromatic proton region of half-filtered 2D H-H NOESY spectra, derived from double-labeled dPHD-HD1 probed with (A) H3R2me2aK4me2 or (B) H3K4me2 (see Figure S5, for full spectra, and protein-only and peptide-only controls that lack cross-peaks in this region). (C and D) HADDOCK model of dPHD-HD1link (same view as in Figure 1C) bound to dually methylated histone H3 peptide, with key residues labeled. See also Figures S5–S7.

To obtain direct evidence for the binding of R2me2a to its groove, we recorded intermolecular ^1^H(^12^C)-^1^H(^13^C) NOEs between ^13^C-^15^N-labeled dPHD-HD1link and unlabeled dually modified histone H3 peptide. We thus resolved 39 intermolecular NOEs, each present in the NOESY spectrum of the ternary complex, but absent from control spectra for dPHD-HD1link alone, or peptide alone (Figure S5), indicating that each NOE reflects a specific H-H contact (<5 Å) between PHD-HD1 and peptide. We were able to assign 35 of these NOEs to specific H-H contacts (Figures S5 and S6), including several contacts between the *N*-methyl groups of K4me2 and R2me2a and aromatic protons of dPHD-HD1 (Figure 5A). The R2me2a-specific contacts were confirmed since only the corresponding peaks (from the proton resonance at 2.977 ppm) disappeared if singly modified peptide was used (Figure 5B). Observation of these R2me2a-specific NOEs is compelling evidence for a direct recognition of R2me2a by the fly PHD-HD1 complex.

Next, we generated a structural model using HADDOCK software (Dominguez et al., 2003; Figure S7): we used the crystal coordinates of dPHD-HD1 (Figure 1B) and histone H3 peptide (residues 1–7, from its complex with hPHD-HD1; Fiedler et al., 2008) as input into HADDOCK, which docks the two input structures while allowing flexible remodeling of the binding interface. Docking was driven by ambiguous restraints from ^15^N-HSQC CSPs, and unambiguous H-H distance restraints from the 35 assigned intermolecular NOEs (Figure S6).

The model reveals that R2me2a is neatly tucked into the R2 groove (Figures 5C and 5D), with close hydrophobic interactions between the two *N*-methyl groups with two protons each (Cε and Cζ) of the phenyl rings of F765 and F773 (2–3 Å, and 3–4 Å, respectively; though see also Figure S6). The side wall opposite F773 is formed by E767 whose negatively charged carboxylate undergoes a close (<2.3 Å) polar interaction with the delocalized positive charge of R2me2a (which is not detectable by NOESY though), exploiting the polarized nature of the R2 groove.

### Histone Binding Triggers Allosteric Modulation of the PHD-HD1 Interface

**Figure 6:**
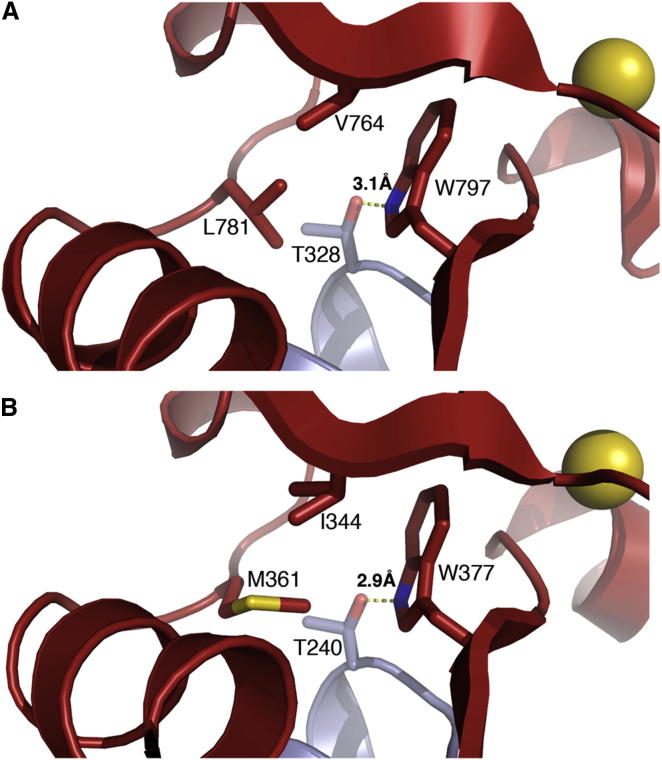
Allosteric Triplet in Fly and Human Pygo PHD Ribbon representations of close-ups of (A) *Drosophila* Pygo or (B) hPygo2; allosteric residues are in stick representations, with key hydrogen bond indicated between PHD signature residue (W) and invariant T of HD1; color-coding as in Figure 1B.

The A1 pocket is buttressed by HD1, whose β1-α1 structure supports the pocket floor. Key for the interaction between PHD and HD1 is a hydrogen bond between an invariant T in HD1 α1 (T328) and W797, the PHD signature residue (Bienz, 2006; Figure 6A). Buttressing of PHD by HD1 is essential for Pygo’s function in development since point mutations in the PHD-HD1 interface abolish Pygo’s binding to Lgs, and its ability to rescue *pygo* null mutants (Kessler et al., 2009; Townsley et al., 2004a).

In the human complex, HD1 binding to PHD triggers a short allosteric mechanism that modulates the shape of its T3 channel, involving hydrophobic interactions between three crucial residues, W377, M361, and I344 (Miller et al., 2010; Figure 6B). The topological equivalent of this allosteric triplet in the fly complex is W797, L781, and V764: W797 and L781 engage in crucial interactions with T328, and also connect with V764, a residue lining the T3 channel (Figure 6A). Thus, the putative allosteric triplet in dPHD (W/L/V) differs from its human counterpart (W/M/I) in two residues that appear structurally adapted to one another.

Intriguingly, histone binding to PHD induces weak CSPs of HD1 residues, notably of F324 (Figure 4B). This effect is significantly stronger with dually-modified peptide (Figure 4D). Indeed, the differential perturbation map highlights the HD1 β strand as the target for this allosteric effect, as well as T328 at the start of the α helix (Figure 4F). Recall that this HD1 section provides buttressing, with T328 stabilizing the A1 pocket floor. Thus, the binding of dually-modified histone H3 tail to fly PHD allosterically modulates its interaction with HD1. Notably, the R2 floor residue is linked directly to V764, the allosteric triplet residue that relays the allosteric effect through the PHD core.

### An Intact Histone-Binding Surface Is Crucial for Pygo’s Function in Tissue Patterning

The lining of the Pygo K4 pocket includes an invariant V (V757, within EVND) whose mutation reduces the histone binding affinity of the human complex, and abolishes the rescue activity of fly Pygo (Fiedler et al., 2008). The same mutant fails to rescue the lethality of *pygo*, in contrast to other point mutants that allow *pygo* mutant embryos to develop into flies (Kessler et al., 2009). However, a Pygo-Lgs chimera in which PHD and HD1 were substituted by a direct link retained only residual rescue activity; >86% of *pygo* mutants expressing this chimera died before adulthood (Kessler et al., 2009), indicating severe dysfunction of PHD-less Pygo. Nevertheless, these results implied that fly development can proceed without histone binding by Pygo.

One caveat is that the design of these mutants was guided by the human PHD-HD1 complex whose structure differs from the fly complex. We thus used the latter to design three mutants: N790E (altering the T3 channel), F765R, and F773R (altering the R2 groove). We expressed these as ^15^N-labeled proteins, to confirm that their folding was normal, as judged by their well-dispersed HSQCs, and that the histone binding of N790E and F773R was abolished, while that of F765R was severely reduced (Figure S8). Importantly, each mutant binds to Lgs as well as wt PHD (Figure S8), reconfirming their structural integrity.

Next, we generated transgenic fly strains expressing these mutants, for Pygo rescue assays. To identify low-expressing lines, we pre-screened our lines with a phenotypic test based on the posterior wing margin, which becomes notched if Pygo is expressed at high levels (Parker et al., 2002). We thus selected several lines without wing notches and confirmed that these express low Pygo levels (Figure S8), including an additional wt line (WT2) whose expression is below that of our standard line (WT1; Fiedler et al., 2008), and monitored their rescue activity in *pygo* null mutant wing disc clones, following GAL4-mediated expression.

**Figure 7:**
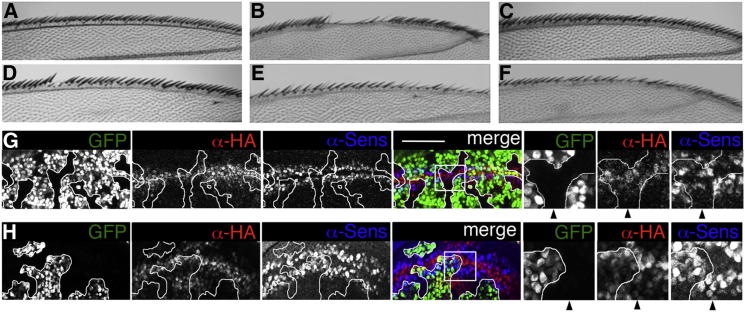
Rescue Assays in Fly Wings (A–F) Anterior wing margins of flies (A) without or (B–F) with *pygo*^*S123*^ mutant clones, (C–F) expressing HA-Pygo; (C) WT2, (D) F765R, (E) N790E, (F) F773R. (G and H) Single confocal sections through late third instar larval wing discs with *pygo*^*S123*^ mutant clones (as traced; marked by absence of GFP, green) expressing (G) N790E or (H) Pygo-gof, stained with α-Sens (blue) and α-HA antibodies (red); scale bar, 25 μm; zoomed areas (boxed in merge) are shown at the right; arrowheads point to *pygo*^*S123*^ mutant clone, revealing (G) partial or (H) full rescue activity (right panels) of (G) N790E or (H) Pygo-gof, and WT1 (Fiedler et al., 2008). See also Figure S8.

*pygo* is required for patterning the wing margin (Figure 7A), and so *pygo* mutant wing disc clones produce margin defects with high penetrance (in 20/22 eclosing flies; Figure 7B; Fiedler et al., 2008; Parker et al., 2002). These are fully rescued by WT2 (n = 46/46; Figure 7C), but not by the mutants: at least half of the N790E- and F773R-expressing flies retain margin defects (36/57 and 22/44, respectively), and even the weaker mutant F765R fails to rescue these defects in a quarter of the flies (9/35; Figures 7D–7F), indicating considerable dysfunction.

We also monitored a transcriptional read-out of Pygo, namely the Wg target *senseless* (*sens*) whose expression is abolished in *pygo* mutant clones (Parker et al., 2002). Expression of *sens* is only partially rescued by N790E (Figure 7G), but is fully rescued by WT2, like WT1 (Fiedler et al., 2008). This confirms that N790E is dysfunctional, similarly to V757E which fails to rescue *sens* expression (Fiedler et al., 2008), demonstrating that the integrity of Pygo’s histone-binding surface is required for normal tissue patterning.

### A Pygo Gain-of-Function Mutant Derepresses Notch Targets

A W > E mutation of the human Pygo1 pocket-divider abolishes histone binding (Fiedler et al., 2008). Likewise, rendering hPygo2 fly-like (by a W > F substitution) reduces, while humanizing fly Pygo (F773W) increases histone binding (Kessler et al., 2009). Indeed, humanized dPHD-HD1 binds robustly to H3K4me2 (K_*d*_ 7.5–11.7 μM), as measured by isothermal calorimetry (ITC; Figure S9), comparable to the human complexes (1.83–3.32 μM, depending on the paralog; Miller et al., 2010). This confirms that the pocket-divider of PHD fingers is a key determinant of their affinity for H3K4me.

Next, we generated transgenic lines expressing F773W (Pygo-gof), to test its effect on tissue patterning. Recall that overexpressed wt Pygo is well tolerated, producing only mild dominant-negative effects in the posterior wing margin (even WT4, expressed at >20× higher levels than WT1; Figure S8). However, Pygo-gof is highly deleterious, causing fully penetrant pupal lethality after wing disc-specific overexpression with *ms1096.GAL4*, which is highly unusual for this GAL4 driver. Peeling the dead flies from their pupal cases revealed rudimentary wings covered in bristles, as caused by hyperactive Armadillo (Riese et al., 1997). They also show excess notal bristles, typically 1–5 additional dorsocentral or postalar bristles per notum (on average 2.9 excess bristles per female; n = 28), similarly to flies expressing LEF-1 (Riese et al., 1997), or constitutively active Armadillo (Arm^S10^; Pai et al., 1997). Thus, Pygo-gof behaves as a hyperactive Wg signaling component.

Importantly, F773W fully rescues *sens* expression in *pygo* mutant wing disc clones (Figure 7H), indistinguishably from WT1. In addition, in the wt territories of these discs, Pygo-gof but not WT4 increases expression of the Wg target *Distalless* (*Dll*) (Neumann and Cohen, 1996) in the prospective wing blade, and overgrowth in the prospective hinge region, similarly to overexpressed Arm^S10^ (Figure S10). Thus, Pygo-gof is fully competent with regard to Wg signaling in the absence of endogenous Pygo, whereas in its presence, Pygo-gof is hyperactive, presumably due to cumulative high expression levels.

Staining polytene salivary gland chromosomes to depict Pygo-associated genomic loci (de la Roche and Bienz, 2007), we found several additional loci for Pygo-gof, suggesting that this mutant might act on novel targets. We focused on Notch targets since Notch signaling precedes Wg expression in the prospective wing margin, and cooperates with Wg in its patterning (Couso et al., 1994; Neumann and Cohen, 1996): *wg* is expressed along the prospective margin in the wing disc (Rulifson and Blair, 1995), in direct response to Notch (Djiane et al., 2013). This is followed by expression of *cut,* another direct Notch target (Djiane et al., 2013; Guss et al., 2001) expressed in a narrow stripe within the Wg signaling zone that defines the prospective margin.

**Figure 8:**
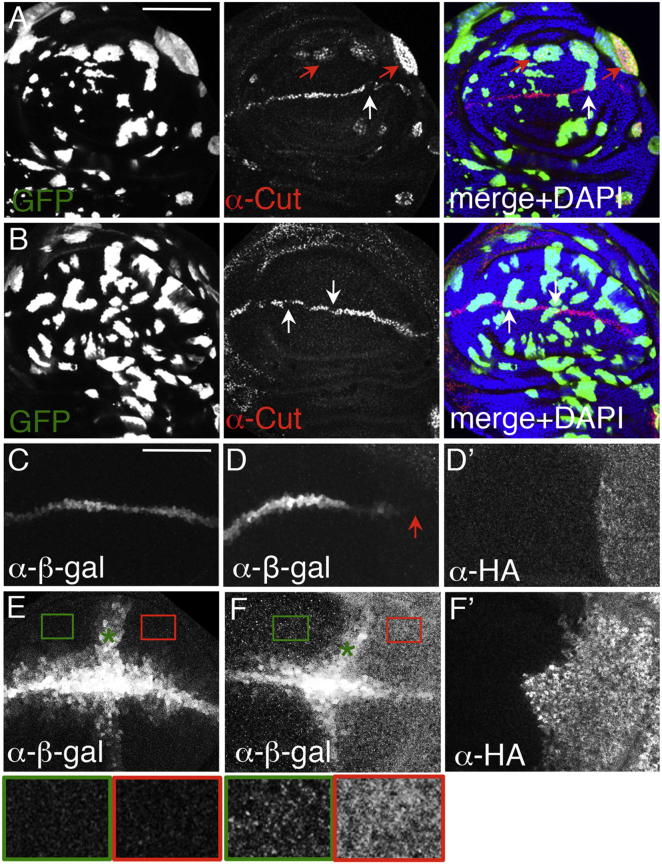
Pygo-gof Derepresses Notch Targets (A and B) Single confocal sections through wing discs as in Figure 7, bearing “flip-on” clones (marked by GFP, green) that express (A) Pygo-gof or (B) WT4, stained with α-Cut antibody (red) and DAPI (blue); red arrows, derepression of *cut* in the prospective hinge; white arrows, repression of normal *cut* expression along the prospective margin. (C–F) Single confocal sections through early third larval instar wing discs, stained for β-galactosidase and HA, to reveal (C and D) *cut-lacZ* or (E and F) *wg-lacZ* reporter activity in cells expressing HA-Pygo-gof (D′ and F′), which represses *cut-lacZ* (red arrow in D) or derepresses *wg-lacZ* (red boxed area in F; asterisks mark *wg-lacZ* reporter activity in cells in which endogenous *wg* is silent); underneath, zoomed views of boxed areas (imaged with identical settings, at the same focal plane). Size bars, (A and B) 40 μm or (C–F) 25 μm. See also Figures S9 and S10.

To test whether Pygo-gof acts on Notch targets, we generated “flip-on” clones of wing disc cells expressing Pygo-gof, and monitored *cut* expression. Strikingly, *cut* is derepressed ectopically and cell-autonomously in a substantial fraction of the flip-on clones (51/95 clones; n = 5 wing discs), predominantly in the prospective hinge, but also near the prospective margin, although within its normal expression domain along the margin itself, *cut* is repressed by Pygo-gof (Figure 8A). Neither ectopic derepression of *cut*, nor its repression along the margin, is seen in flip-on clones of WT1, nor of Arm^S10^ (Figure S10). Even the super-high expressing WT4 line does not derepress *cut* ectopically (though in this case, we observe *cut* repression along the margin; Figure 8B). Thus, Pygo-gof derepresses a Notch target that is unresponsive to ectopic Wg—a striking illustration of the gain-of-function of this humanized mutant.

To consolidate this, we monitored the effects of Pygo-gof on two minimal Notch-responsive enhancers from the upstream regions of *cut* and *wg* (*cut-lacZ* and *wg-lacZ*; Djiane et al., 2013), following Pygo-gof expression in the posterior compartment. *cut-lacZ* recapitulates endogenous *cut* expression (Figure 8C), and is repressed by Pygo-gof (Figure 8D, arrow), although it is not ectopically activated like endogenous *cut*, likely because the *hsp70* promoter in the *cut-lacZ* reporter does not respond to remote silencers and enhancers (Müller and Bienz, 1991). *wg-lacZ* also mimics endogenous *wg* expression along the margin (Figure 8E), and is derepressed by Pygo-gof throughout the posterior territory (Figure 8F). No ectopic activation is seen with WT4, reinforcing our conclusion that Pygo-gof is a genuine gain-of-function mutant acting on Notch targets.

## Discussion

### Evolution of a Deviant Histone-Binding Surface in Fly Pygo PHD Fingers

PHD fingers from brachycera Pygo orthologs are distinguished from classical PHD fingers by a W > F substitution in their pocket-dividers, which is also found in the Pygo ortholog of the nematode *Prionculus punctatus* (Figure S1). While this does not abolish the semi-aromatic character of the K4 pocket that determines its reliance on methylated K4 (Taverna et al., 2007), it widens the T3 channel. This is compensated for (at least partially) by a T > N substitution narrowing the channel from the opposite side (T > K in the medfly, *Ceratitis capitata*). These two substitutions allowed the R2 groove to evolve as a distinct structural feature. Their covariance in brachycera and their compensatory structural consequences indicate a functional divergence rather than a random mutational drift leading to loss-of-function. Notably, T > N requires only a single codon base mutation, while W > F requires two, arguing that T > N may have been the primary mutation, with W > F occurring secondarily. The latter could have created a bottleneck during evolution, perhaps explaining its rarity (so far only found in two animal phyla).

### Interaction of R2me2a with Its Cognate Groove in *Drosophila* Pygo PHD

Notably, the R2 groove floor involves a third substitution (L > F) which, again, requires a single codon mutation. This causes a significant change of the R2me2-cognate binding surface because it removes the hydrophobic L side-chain that fills most of the R2 cavity in classical Pygo orthologs, exposing the terminal guanidium group of R2 and conferring indifference to R2 methylation (Fiedler et al., 2008; Miller et al., 2010). Indeed, the shallow R2 groove in fly Pygo PHD is reminiscent of classical PHD fingers whose deep R2 cavity buries the R2 guanidinium group (e.g., Peña et al., 2006), although some of these PHD fingers cannot accommodate methylated R2 (Kirmizis et al., 2007; Vermeulen et al., 2007). Thus, the mode of R2 recognition varies considerably among PHD fingers, burying unmodified R2 and potentially incompatible with methylated R2 (in classical H3K4me-interacting PHD fingers), exposing the side-chain of R2 and conferring indifference to R2 methylation (in classical Pygo orthologs), or embedding dimethylated R2 (in fly Pygo).

The semi-aromatic R2 groove mirrors the adjacent semi-aromatic K4 pocket with which it shares F773 as a side wall. This residue undergoes direct hydrophobic interactions with one of the two methyl groups of R2me2a, as shown by our NMR data, which also indicate close hydrophobic contacts between the other methyl group of R2me2a and the R2 groove floor (F765). By contrast, the side wall opposite F773 is formed by E767 whose carboxylate, according to our structural model, undergoes a close polar interaction with the de-localized positive charge of R2me2a. This differs from Tudor domains (the only structural precedents with pockets embedding Rme2a) whose fully aromatic cages undergo cation-π stacking interactions with Rme2, allowing flexible accommodation of both symmetric and asymmetric arginine methylations (Tripsianes et al., 2011). Thus, the PHD finger of fly Pygo exhibits a distinct mode of recognizing dimethylarginine.

Our model indicates that Pygo simultaneously recognizes two chromatin marks—histone H3 tail methylated at both R2 and K4. To our knowledge, there is only one report of a PHD finger recognizing dually modified histone H3 tail, namely that of RAG2: the topological equivalent of F765 in RAG2 is a tyrosine whose phenyl ring was proposed to interact with R2me2a (Ramón-Maiques et al., 2007), although this has not been confirmed by structural analysis. The ability of the *Drosophila* Pygo-Lgs complex to recognize R2me2a-modified histone H3 has interesting functional implications since this chromatin mark is associated with silenced loci (Guccione et al., 2007; Kirmizis et al., 2007): it implies that Pygo recognizes repressive chromatin, consistent with its association with repressed Wg target genes (de la Roche and Bienz, 2007) and its antirepressor role in relieving Groucho-mediated repression of Wg targets during incipient Wg signaling (Mieszczanek et al., 2008).

### Allosteric Communication between Lgs- and Histone-Binding PHD Surfaces

Our NMR analysis revealed that the β strand residues of HD1 experiences the remote binding of histone H3 tail to the opposite surface of fly PHD. Evidently, the two surfaces of this PHD finger communicate via a short allosteric mechanism relayed through its structural core. The dually modified histone tail is more effective in modulating the HD1 surface, which could increase Pygo’s affinity for Lgs and, consequently, facilitate the recruitment of Armadillo to silenced Wnt targets. This echoes the situation in mouse Pygo2 whose binding to histone H3 tail increases its affinity to BCL9 (Gu et al., 2009). Both occur in the reverse direction to that previously described for human PHD (Miller et al., 2010), consistent with the intrinsic bidirectionality of allosteric effects. Either, or both, could be functionally relevant.

Relaying the allosteric communication through the structural PHD core relies on finely-tuned interactions, mostly between three key residues including the invariant PHD signature W (Miller et al., 2010). Interestingly, the other two residues of the allosteric triplet differ between fly and human, involving substitutions (I > V and M > L) that are structurally adapted to one another, each requiring only a single codon base change, possibly reflecting yet another functional coadaption. The structural connectivity between the two PHD surfaces (which involves the R2 groove) may have been preserved in response to selective pressure.

### Physiological Relevance of Pygo’s Chromatin Binding

A previous study questioned the physiogical relevance of Pygo’s histone binding (Kessler et al., 2009), which we refute as follows. First, the compromised rescue activity of four different Pygo mutants indicate that the structural integrity of the histone-binding surface is crucial for normal tissue patterning (Figure 7; Fiedler et al., 2008; Kessler et al., 2009). Second, the evolution of the R2 groove as a distinctstructural feature in fly Pygo PHD, and the preservation of the complex allosteric connectivity between its two ligand-binding surfaces argue that the underlying amino acid substitutions occurred in response to selective pressure. Notably, histone binding could have been lost at random by numerous single mutations, e.g., in the pivotal W pocket-divider, which can mutate, by seven single codon substitutions, to five amino acids (C, R, G, L, or S) several of which would preserve the structural integrity of the PHD finger and its Lgs binding, so should be well tolerated. Recall though that the crucial W > F substitution found in flies requires two codon base mutations, unlikely the result from random drift.

The histone binding affinity of Pygo is clearly too weak to confer its recruitment to Wg targets, although its PHD finger might synergize with its conserved NPF motif in this process (de la Roche and Bienz, 2007). Indeed, PHD fingers in other proteins synergize with linked domains (e.g., bromo domains) in the binding to chromatin (Taverna et al., 2007). Alternatively, Pygo’s PHD finger might scan for cognate histone H3 marks associated with silenced Wnt targets and, by binding to them, acquire a higher affinity for Lgs (through allosteric modulation), which would facilitate the efficient capture of Armadillo during incipient Wg signaling (de la Roche and Bienz, 2007; Mieszczanek et al., 2008; Townsley et al., 2004b). However, given that the R2me2a is firmly tucked into its cognate groove, we favor the possibility that Pygo could attenuate its demethylation, thus delaying the derepression of Wg targets during incipient Wg signaling. Importantly, since Pygo is primarily associated with chromatin through its NPF ligand (de la Roche and Bienz, 2007), its binding to histone H3 tail is tantamount to an intramolecular interaction within a pre-formed multi-protein complex, which requires far lower affinities than intermolecular protein-protein interactions aimed at complex formation.

Importantly, low yet functionally relevant binding affinities are widespread among signaling molecules. Indeed, individual Tudor domains have a low binding affinity for Rme (Tripsianes et al., 2011) or Kme (Huang et al., 2006), but this is increased significantly by tandem linkage of multiple domains in the same protein. Low binding affinities are desirable for dynamic protein interactions requiring combinatorial recognition, and can be overcome by multimerization and clustering of interacting modules, which produces a high local concentration (e.g., in Wnt signalosomes) and enhances the avidity for low-affinity ligands (Schwarz-Romond et al., 2007).

While this manuscript was under revision, Cantu et al. published evidence arguing against the physiological relevance of Pygo’s histone binding during mouse development, based on a knock-in of a histone binding-deficient Pygo2 mutant (Cantù et al., 2013). Unfortunately, this mutant (A342E, identical to A356E in hPygo1) retains significant histone binding (K_*d*_ = 109 ± 8 μM; Fiedler et al., 2008), with an approximate ten times higher affinity than fly Pygo, which therefore seriously undermines the conclusions drawn by these authors (Cantù et al., 2013).

### An Inherent Activity of Pygo in Derepressing Notch Targets

Humanizing fly PHD increased its histone-binding affinity, but proved deleterious to fly development, possibly because this enabled Pygo to derepress Notch-dependent transcription. Notably, Notch targets are subject to Groucho-dependent repression, like Wg targets (Jennings and Ish-Horowicz, 2008). It is thus tempting to speculate that Pygo’s low histone-binding affinity suffices for relieving Groucho-dependent repression of Wg targets, while relieving that of Notch targets requires a far higher affinity. Regardless, the altered histone-binding surface of the fly PHD finger seems an evolutionary adaptation of Pygo to lose its antirepressor activity toward Notch but not Wg targets (Mieszczanek et al., 2008). This adaptation may have occurred multiple times in evolution, given the appearance of the W > F substitution in a nematode Pygo ortholog. It will be interesting to see whether this substitution is also present in other animal phyla.

An interesting corollary of our Pygo-gof results is that classical Pygo orthologs may have an intrinsic activity of derepressing Notch targets. If so, this would explain why *Pygo* knock-out mice show Wnt-unrelated phenotypes in addition to Wnt-related defects (Li et al., 2007; Schwab et al., 2007; Song et al., 2007). Notch and Wnt signaling cooperate in patterning intestinal crypts in mice, including the stem and progenitor cells for intestinal homeostasis and regeneration (Buske et al., 2011; Radtke and Clevers, 2005), and the cells-of-origin for intestinal neoplasia (Barker et al., 2009). Further analysis will be required to test whether human Pygo mediates the synergy between these two signaling pathways in the intestine.

## Experimental Procedures

### Protein Purification

For crystallography, *Drosophila* Pygo PHD (amino acids 747–808) fused to GST, and Lgs HD1 (amino acids 321–355) fused to MBP, were coexpressed with a bicistronic expression vector (including N-terminal TEV protease sites for removal of tags) in *Escherichia coli* BL21-CodonPlus(DE3)-RIL cells (Stratagene), and PHD-HD1 complexes were purified as described (Fiedler et al., 2008). dPHD-HD1link (amino acids 744–803 and 321–352, respectively, separated by GSGSGSG, cloned in pETM11) was expressed similarly, purified by Ni-NTA resin and size exclusion chromatography, and the His tag was removed for NMR (Miller et al., 2010).

### X-Ray Crystallography

Concentrated protein was centrifuged at 100,000 × *g* for 15 min and used for crystallization as described (Fiedler et al., 2008; initial screen of ∼1,500 different crystallization conditions in 100 nl drops in a 96-well sitting-drop format). Crystals emerged under multiple conditions after growing for several days at 19°C by the vapor diffusion method, and were soaked for < 1 min in 25% glycerol as cryo-protectant before flash-cooling in liquid nitrogen. X-ray diffraction data were collected at 100 K with an ADSC detector using the ESRF Grenoble synchrotron ID23-1 beamline, from crystals grown in 1.136 M (NH_4_)_2_SO_4_, 100 mM Tris pH 8.3, and 200 mM NaCl, and the data were processed as described in Figure S2. Structural images were drawn with PyMol.

### NMR Spectroscopy

[^1^H,^15^N]fast-HSQC spectra (Mori et al., 1995) were recorded on a Bruker Avance III spectrometer operating at 600 MHz ^1^H frequency, with a 5 mm cryogenic inverse probe (sample temperature 293 K). Spectra were acquired with 1,024 points in t_2_, and 128 complex points in t_1_ extended to 256 by forward linear prediction. The digital resolution of processed data was 0.7 Hz/point and 1.0 Hz/point in *f*_2_ and *f*_1_, respectively. Spectra were processed with TopSpin version 3.0 (Bruker) and analyzed with Sparky version 3.113 (Goddard and Kneller, UCSF). Backbone resonances were assigned with standard triple resonance correlation spectra (HNCACB, CBCA(CO)NH, HNCO and HN(CA)CO), using unmodified Bruker pulse programs. Side chain resonances of the ternary complex were assigned using (H)CC(CO)NH, H(CCCO)NH and 2D [^1^H,^13^C]-HSQC. Resonances of the histone peptide were assigned from an unedited [^1^H, ^1^H]-TOCSY of the complex in a 95% H_2_O, 5% D_2_O buffer containing 25 mM phosphate, and 150 mM NaCl (pH 6.7). A partial assignment of aromatic ^1^H resonances was obtained from (HB)CB(CGCD)HD and (HB)CB(CGCDCE)HE spectra (Yamazaki et al., 1993). The identity and bonding of *N*-methyl groups of H3R2me2aK4me2 were confirmed with three-bond ^1^H-^13^C correlations in 2D HMBC spectra of peptide alone. ω_1_-^13^C-filtered-ω_2_-^13^C-edited NOESY spectra (Otting and Wuthrich, 1989) were acquired with X half-filters set to accept only cross-peaks between ^12^C-coupled protons and ^13^C-coupled protons, using a pulse program employed previously (Eustermann et al., 2011), and recorded on a Bruker 800 MHz Avance I spectrometer, with cryogenic inverse probe, using a sample as above but dialyzed into D_2_O buffer, and an NOE mixing time of 150 ms.

### HADDOCK Calculations

Simulations were performed with HADDOCK version 2.1 (Dominguez et al., 2003) using CNS version 1.3 (Brünger et al., 1998). HADDOCK was implemented with default settings, except for retention of nonpolar hydrogen atoms throughout. For the R2me2a side-chain, the partial charges, geometry and tautomer were implemented as described (Tripsianes et al., 2011). Docking was guided by ambiguous restaints derived from [^1^H,^15^N]fHSQC CSP, and unambiguous restraints derived from intermolecular ^1^H(^12^C)-^1^H(^13^C) NOEs (Figure S6). Unambiguous NOE distance restraints were applied as a symmetric biharmonic potential without penalty in the distance range 1.8–3.6 Å, or 1.8–4.7 Å, according to the intensity of NOE cross peak. For the final models, 200 structures were refined with explicit water, all of which occupied a single cluster with no NOE violations, no Ramachandran dihedral violations, and no noncovalent van der Waals conflicts as judged by PROCHECK.

### Fly Assays

HA-Pygo mutants were inserted into pUAST, and independent transformants were isolated as described (Townsley et al., 2004a). Standard transposon mobilization was used to generate new wt Pygo lines. At least two lines were tested for rescue activity in *pygo* null mutant wing disc clones (generated with *vg.GAL4 UAS.flp*; Végh and Basler, 2003) as described (de la Roche and Bienz, 2007; Fiedler et al., 2008). Other GAL4 drivers used for overexpressing HA-Pygo in wt tissue (*en.GAL4, ms1096.GAL4*) are described in Flybase. Paraformaldehyde-fixed discs were stained with α-Sens (Nolo et al., 2000), α-Dll (Neumann and Cohen, 1996), α-Cut (Developmental Studies Hybridoma Bank), α-β-galactosidase (Promega), or α-HA (Roche) as described (Fiedler et al., 2008; Parker et al., 2002). All discs were counter-stained with DAPI, to control for the focal plane, and single confocal images were acquired at identical settings with a Zeiss Confocal Microscope. Notch-responsive enhancers from *cut* and *wg* have been described (Djiane et al., 2013; A. Djiane and S. Bray, personal communication).

## Figures and Tables

**Table 1:** Data Collection and Refinement Statistics

Data Collection	
Strategy	125°, Δϕ 0.5°
Wavelength	1.2843
Space group	P2_1_2_1_2_1_
a, b, c (Å)	105.21, 111.96, 190.76
α, β, γ (°)	90.0, 90.0, 90.0
Resolution (Å)^a^	46.24–2.68 (2.82–2.68)
Rmerge (%)^b^	14.2 (61.2)
Mean I/σ(I)	8.7 (2.2)
Completeness (%)	99.7 (100)
Multiplicity	5.0 (5.1)
Complexes in A.U.	18

**Refinement**	

Resolution (Å)	46.24–2.68 (2.75–2.68)
No. of reflections	60454
Test set size (%)	5.1
Rwork (%)	22.4 (33.2)
Rfree (%)	26.2 (37.1)
No. atoms (non-H)	14,064
Residues (PHD/HD1)	743–804/312–343
<B > (Å^2^)	40.8
Rmsds	
Bond length (Å)	0.012
Bond angle (°)	1.597
Ramachandran plot	
In favored regions (%)	97.5
Outliers (%)	0
Molprobity clash score	7.79 (99^th^ percentile)

See also Figure S2.

a Highest resolution shell (in Å) shown in parentheses.

b Rmerge = Σ_hkl_ |I_hkl_ − < I_hkl_ > | / Σ_hkl_ I_hkl_.

## Accession Numbers

The Protein Data Bank accession code for the coordinates and structure factors reported in this paper is 3ZPV.
