# Supplementary material for: Evolutionary Adaptation of the Fly Pygo PHD Finger toward Recognizing Histone H3 Tail Methylated at Arginine 2
Source: Structure. 2013 Dec 3;21(12):2208–20. doi: 10.1016/j.str.2013.09.013 (PMC3851687; doi:10.1016/j.str.2013.09.013)
Supplement: Document S1. Figures S1–S10 [file mmc1.pdf]

Structure, Volume *21*

Supplemental Information

Evolutionary Adaptation of the Fly Pygo PHD Finger toward Recognizing Histone H3 Tail Methylated at Arginine 2

Thomas C.R. Miller, Juliusz Mieszczanek, María José Sánchez-Barrena, Trevor J. Rutherford, Marc Fiedler, and Mariann Bienz

Species	Start	Sequence	End
human Pygo1	340	VYPCGICTN**EVND**DQDAILCEASCQKWFHRICTGMTETAYGLLTAEASAVWGCDTCMADKDVQLM	404
human Pygo2	327	VYPCGACRS**EVND**DQDAILCEASCQKWFHRECTGMTESAYGLLTTEASAVWACDLCLKTKEIQSV	391
Xenopus laevis Pygo2	310	IYPCGACER**EVND**DQDAILCEASCQKWFHRECTGMTESAYSLLTREVSAVWACDYCLKTKDIQSV	374
Danio rerio Pygo2	492	VFPCGLCMS**EVHD**DQEAILCEASCQRWFHRDCTGLTEPAYGLLTRESAAVWACDFCLKTKEIQAV	556
Ciona intestinalis	286	PQTCCNCSND**I**QPTDDAIRCLASCNKWFHRTCVGLTESACNFLRSEELALWACDNCLKTKEINSV	350
Strongylocentrotus purpuratus	152	LFPCGICHQQ**V**Q**D**SEDAVICVSSCHTWFHRICTGMTTTAYTLLNSEHAAEWVCDRCVREKKIPLV	216
Trichuris muris	94	ICPCGKCHR**EIHD**NDQAIQCYRGCKFWFHRTCVGLVEEAWHMIVNEPYAEWVCDACLVAKQIPFV	158
Trichinella spiralis	119	ICPCGKCHR**EIHD**NDQAIQCYRGCKFWFHRTCVGLLEEAWHMVINEPYAEWVCDSCLATKQIPCV	183
Metaseiulus occidentalis	70	MYPCGVCHK**EVHD**NDQAVFCEPGCNFWFHRICTGLTEAAFHMLTQEIAAEWVCDKCAVHNKVPLV	134
Tribolium castaneum	242	IYPCGVCHK**EVHD**NDQAILCESGCNFWFHRGCTGLTEAAFQLLTAEVYAEWVCDKCLSSKNIPLV	306
Solenopsis invicta	433	IYPCGICHK**EVHD**NDQAILCESGCNFWFHRSCTGLTEYAYQLLTAEVYAEWVCDKCLQSKSIPLV	497
Acyrthosiphon pisum	437	IYPCGNCHK**EVHD**NDQAVLCESGCNFWFHRVCTGLMEPAFQLLTAEVYAEWVCDKCLQTKNIPLI	501
Camponotus floridanus	419	IYPCGVCHK**EVHD**NDQAILCESGCNFWFHRGCTGLSEHAFQLLTAEVYAEWVCDKCLNSKNIPLV	485
Anopheles gambiae	819	IYPCGGCHK**EVHD**NDQGILCESGCNFWFHRTCSGLTEAAFNLIHAEVYAEWCCDKCLNSKNIPLV	883
Danaus plexippus	424	IYPCGVCHK**EVHD**NDQAILCESGCNFWFHRGCTGLTEPAFQLLTAEVYAEWVCDKCLHSKNIPLV	488
Ixodes scapularis	146	IYPCGVCHK**EVHD**NDQAILCESGCNFWFHRICTGLTDAAFHLLTQEVYAEWVCDRCLSSKSIPLV	210
Hydra magnipapillata	133	IYPCGICTK**EV**S**D**TDEAILCEAGCEFWYHRSCTGMTDIAYQLLTNQDNAEWVCDKCIATKSVPLV	197
@Nematostella vectensis	6	IYPCGICQK**EVND**DDDAILCETGCGRWYHRVCTGLTIMAYNLLTAETSAEWVCNSCIESKNIPLV	70
@Amphimedon queenslandica	25	DFPCGICFE**EV**K**D**EDEGILCESGCDKWYHRQCAGMSKNAYDLLTREDSAEWACDTCIKKNNIPMT	89
@Mnemiopsis leidyi	19	DYPCGICDE**EV**G**D**NDDGIFCESGCDLWYHRTCTGMTTDAYKLLTTEVDAEWACDNCIVRGGVKLV	83
@Trichoplax adhaerens	44	SYPCGACGK**EVND**NDDAILCESGCDVWFHRACTGLSQSAYGYLTSEENAEWICDNCYTSKAIPLT	108
Drosophila Pygo	747	IYPCGMCHK**EVND**NDEAVFCESGCNFFFHRTCVGLTEAAFQMLNKEVFAEWCCDKCVSSKHIPMV	811
Prionchulus punctatus	84	ICPCGVCHI**EIHD**NEQALHCMCGCKFFFHRSCVGMTEEAFNLISNEPLAEWICDNCLTQKQIPPV	148

Zn^{++} coordinating residues C_4HC_3

Pygo signature EV/IN/HD

@ N-terminal sequence not known

Pocket divider

R2 binding site

Allosteric triplet

T3 Channel (south wall)

Figure S1

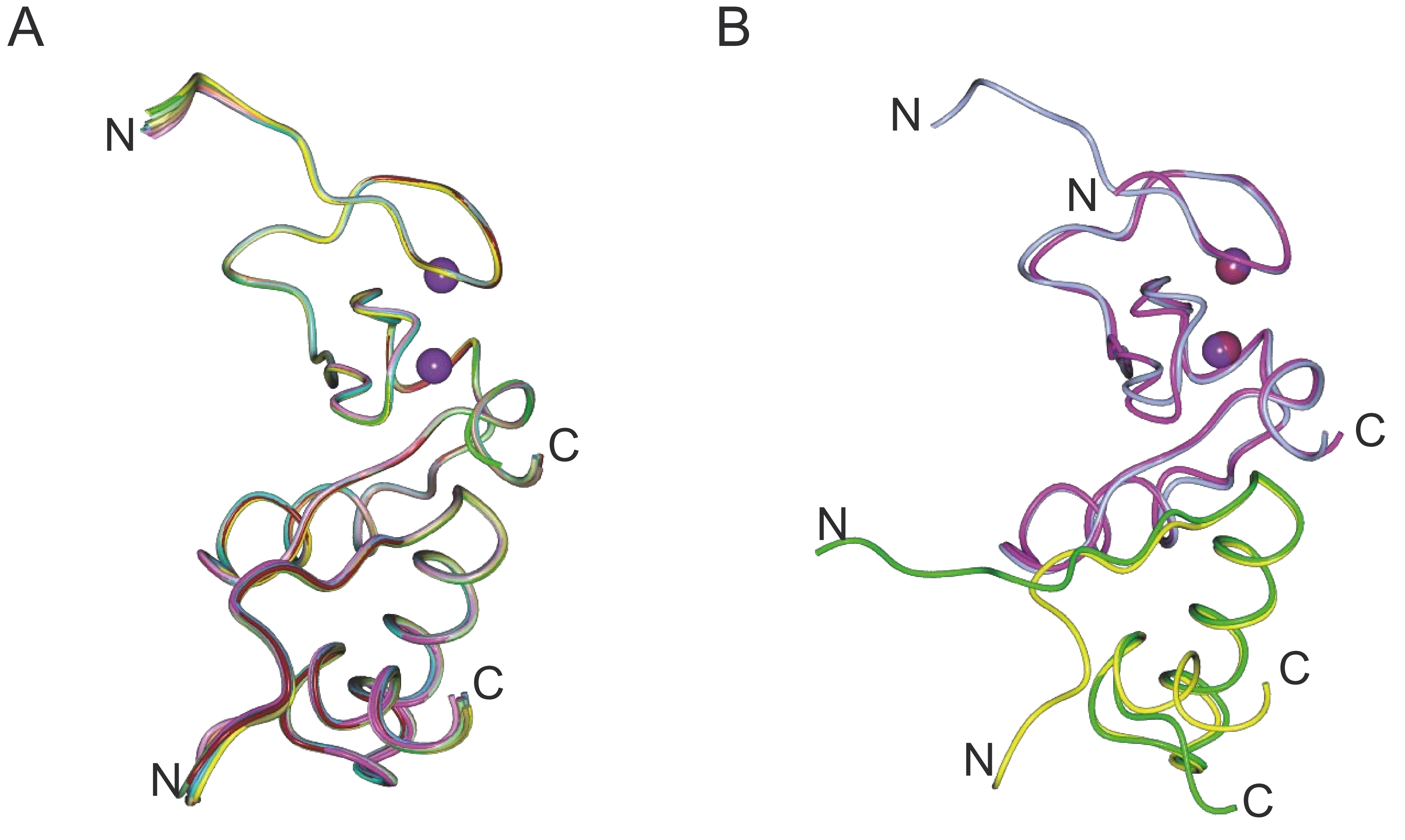

Figure S2

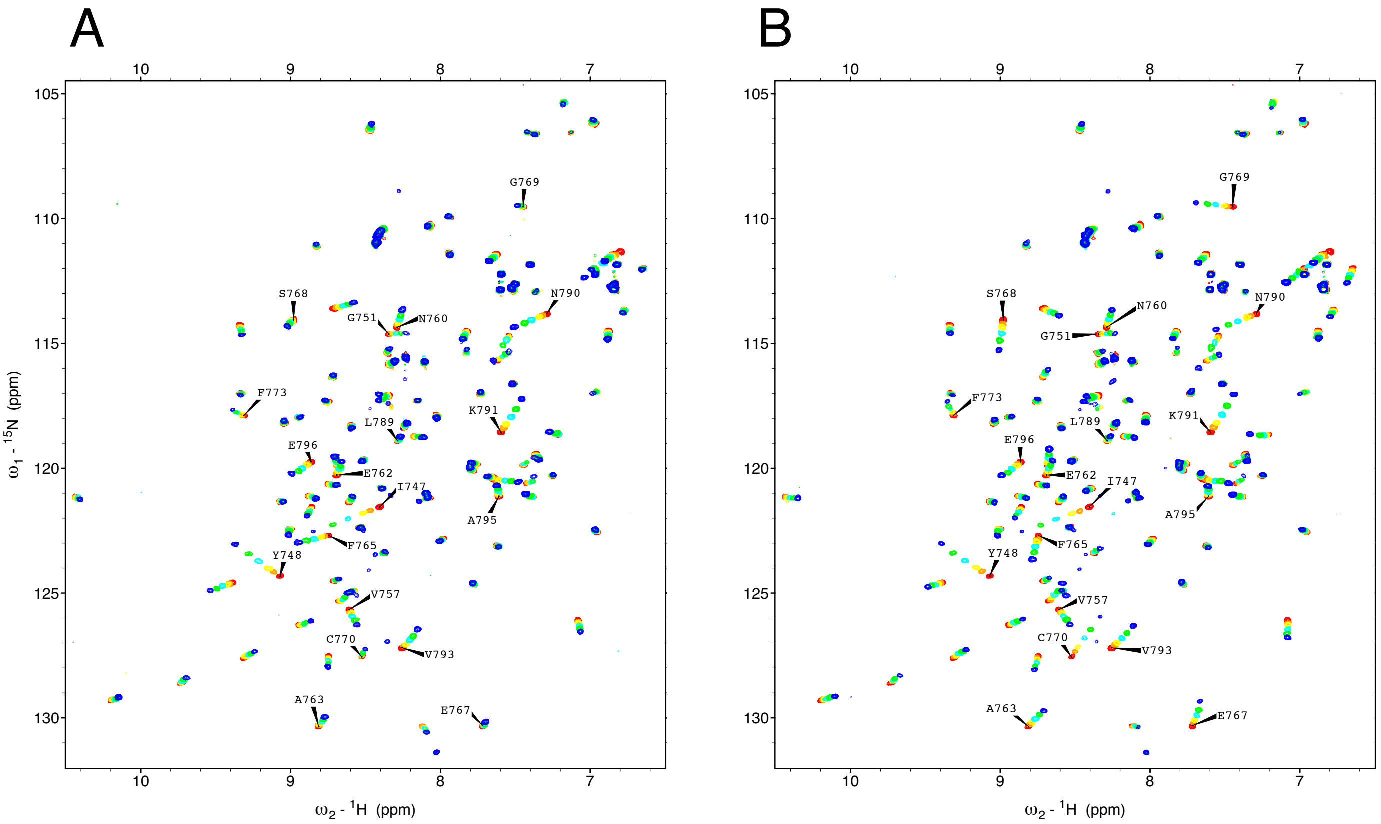

C

	H3R2me2aK4me2		H3K4me2	
	K_d (µM)	std err	K_d (µM)	std err
**I747**	1069	18	1032	40
**Y748**	941	28	1100	25
**G751**	922	100	1117	118
**V757**	1206	94	1290	109
**N760**	1178	74	1093	96
**E762**	1110	113	1019	167
**A763**	1083	113	994	87
**F765**	1111	74	875	31
E767	1570	156	NAN	
S768	1347	95	3252	507
G769	1327	110	3305	1654
C770	1235	56	3314	877
F773	1178	163	830	148
L789	844	201	3068	1265
**N790**	1293	56	1291	60
**K791**	1193	40	1178	38
**V793**	1472	55	1058	46
**A795**	983	139	844	85
**E796**	1283	88	1222	141
Median	1197 +/- 275 µM		1068 +/- 223 µM	

D

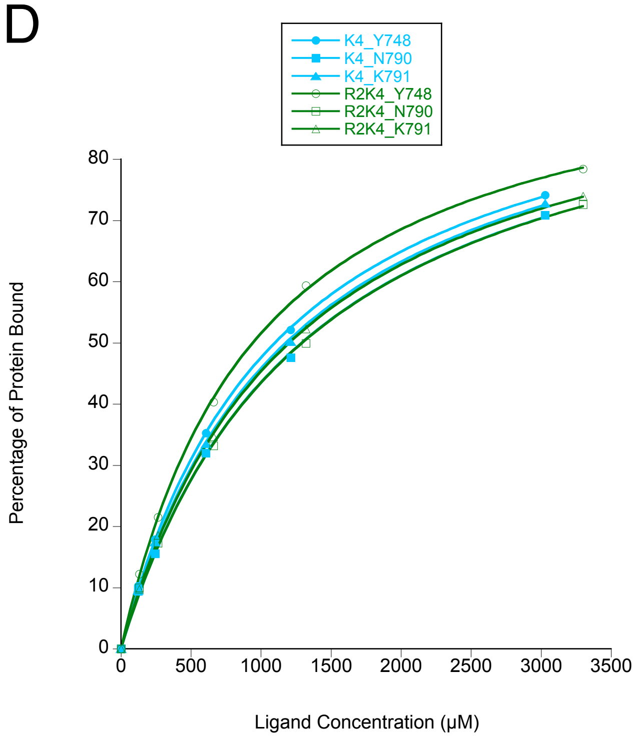

Figure S3

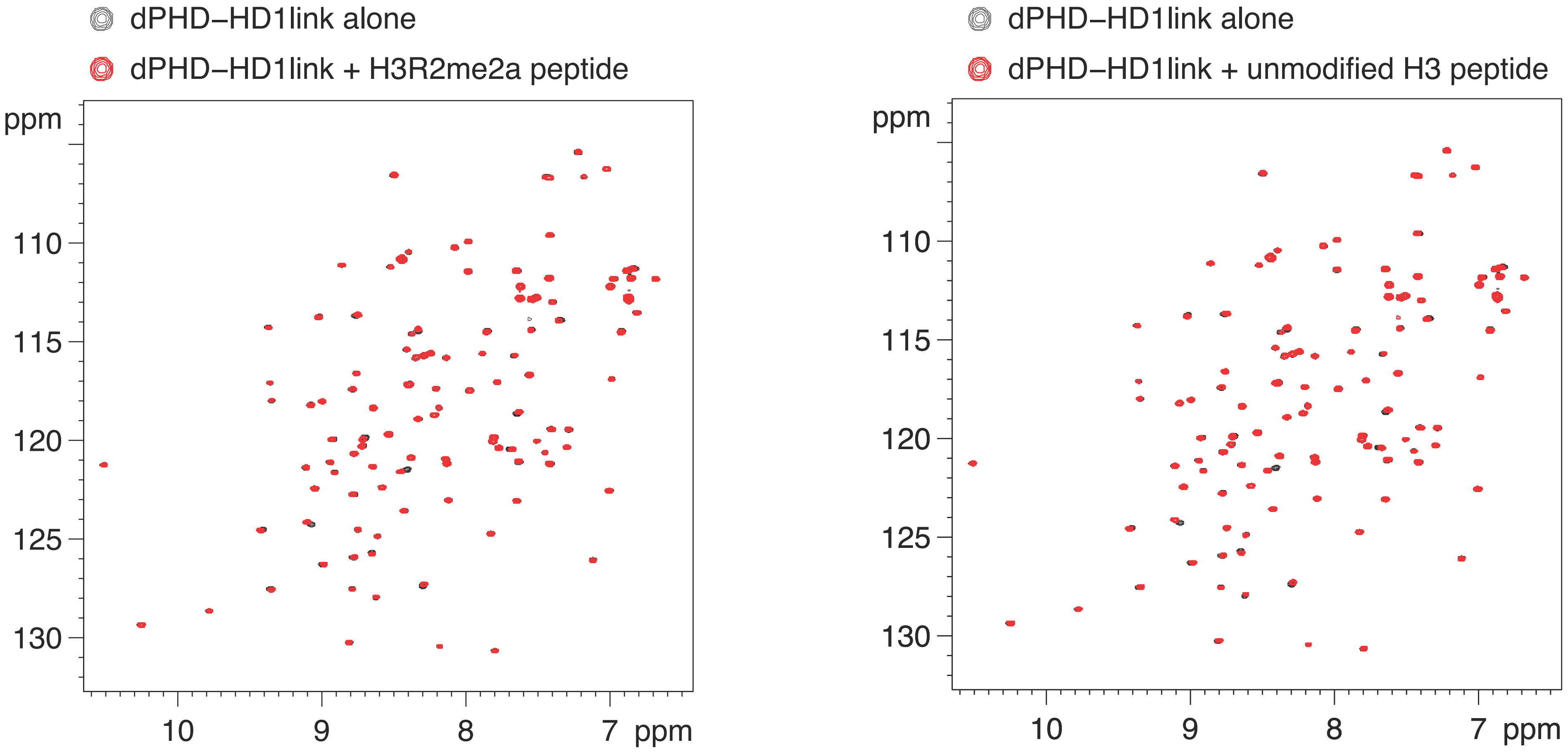
dPHD−HD1link alone
dPHD−HD1link + H3R2me2a peptide
ppm
110
115
120
125
130
10
9
8
7 ppm
dPHD−HD1link alone
dPHD−HD1link + unmodified H3 peptide
ppm
110
115
120
125
130
10
9
8
7 ppm

Figure S4

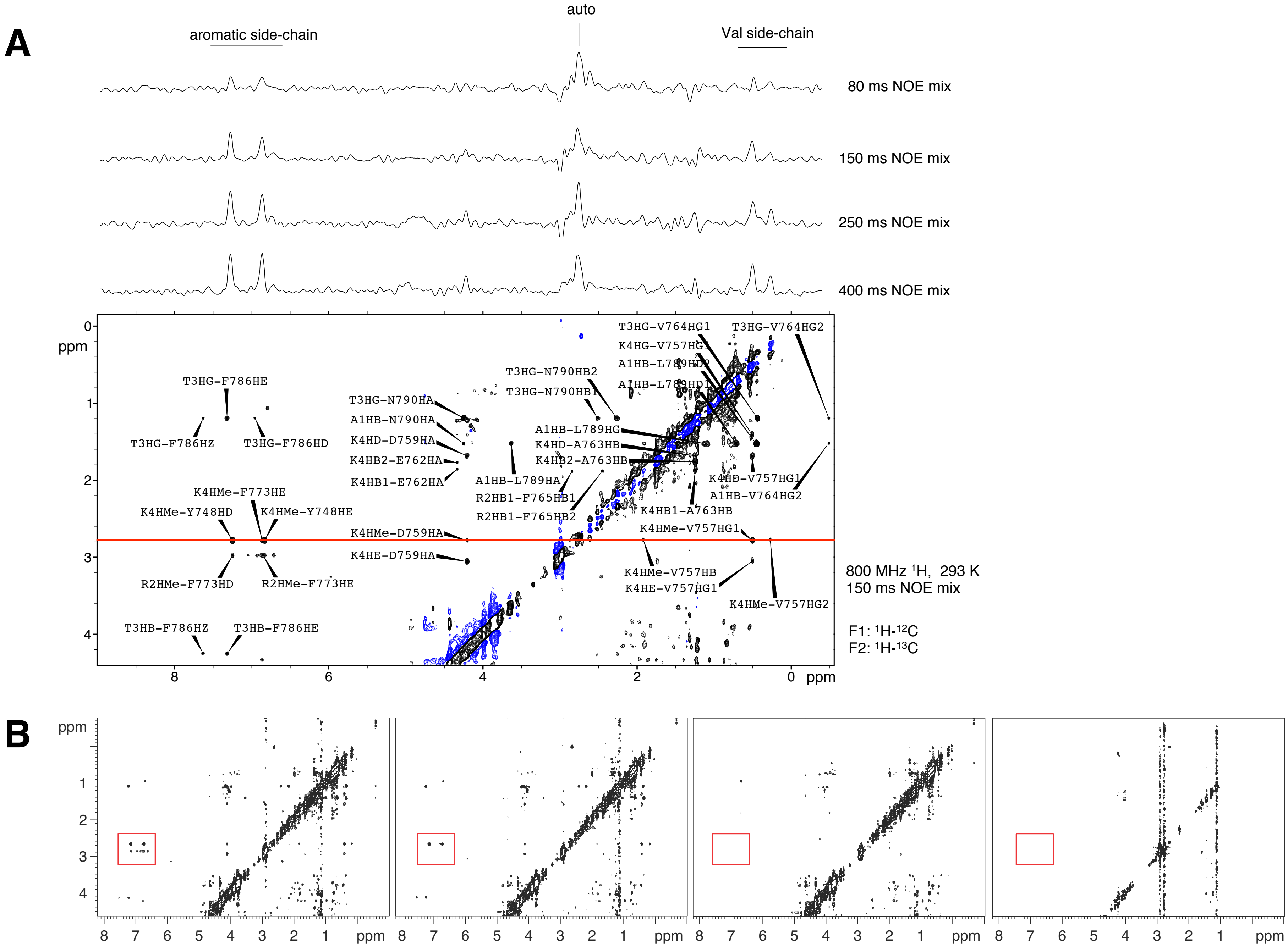
A
auto
aromatic side-chain
Val side-chain
80 ms NOE mix
150 ms NOE mix
250 ms NOE mix
400 ms NOE mix
T3HG-V764HG1
T3HG-V764HG2
K4HG-V757HG1
A1HB-L789HD2
A1HB-L789HD1
T3HG-N790HB2
T3HG-N790HB1
T3HG-F786HE
T3HG-N790HA
A1HB-N790HA
K4HD-D759HA
K4HB2-E762HA
K4HB1-E762HA
T3HG-F786HZ
T3HG-F786HD
A1HB-L789HG
K4HD-A763HB
K4HB2-A763HB
A1HB-L789HA
R2HB1-F765HB1
R2HB1-F765HB2
K4HD-V757HG1
A1HB-V764HG2
K4HB1-A763HB
K4HMe-F773HE
K4HMe-Y748HD
K4HMe-Y748HE
K4HMe-D759HA
K4HMe-V757HG1
K4HE-D759HA
R2HMe-F773HD
R2HMe-F773HE
K4HMe-V757HB
K4HE-V757HG1
K4HMe-V757HG2
T3HB-F786HZ
T3HB-F786HE
800 MHz ¹H, 293 K
150 ms NOE mix
F1: ¹H-¹²C
F2: ¹H-¹³C
ppm
B

Figure S5

Unambigous restraints from H-H intermolecular NOE

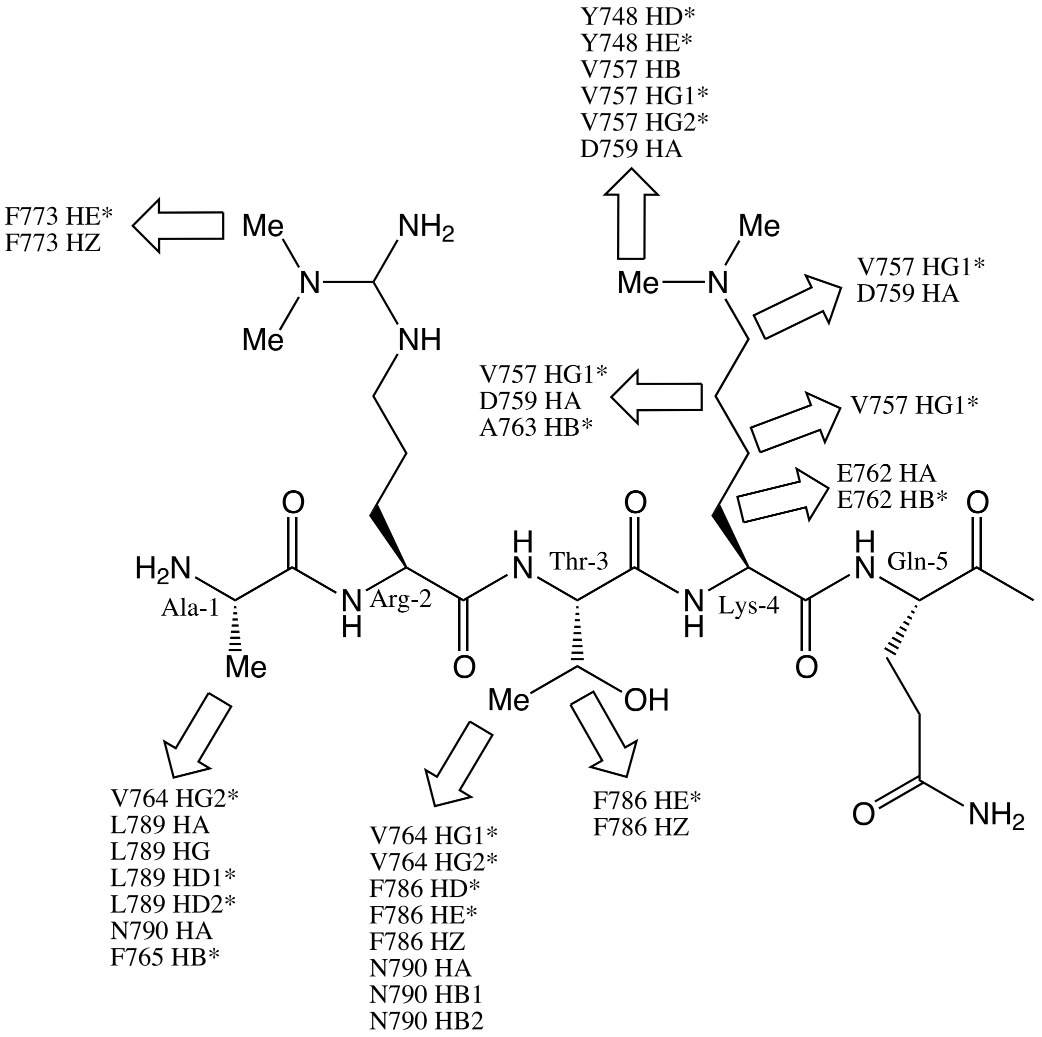

Ambiguous restraints from chemical shift perturbation

active residues (15): 351, 747, 748, 758, 760-3, 767-9, 773, 790, 791, 793

passive residues (8): 323, 325, 352, 756, 771, 772, 794, 795

active residues for peptide (5): 1-5

Figure S6

A

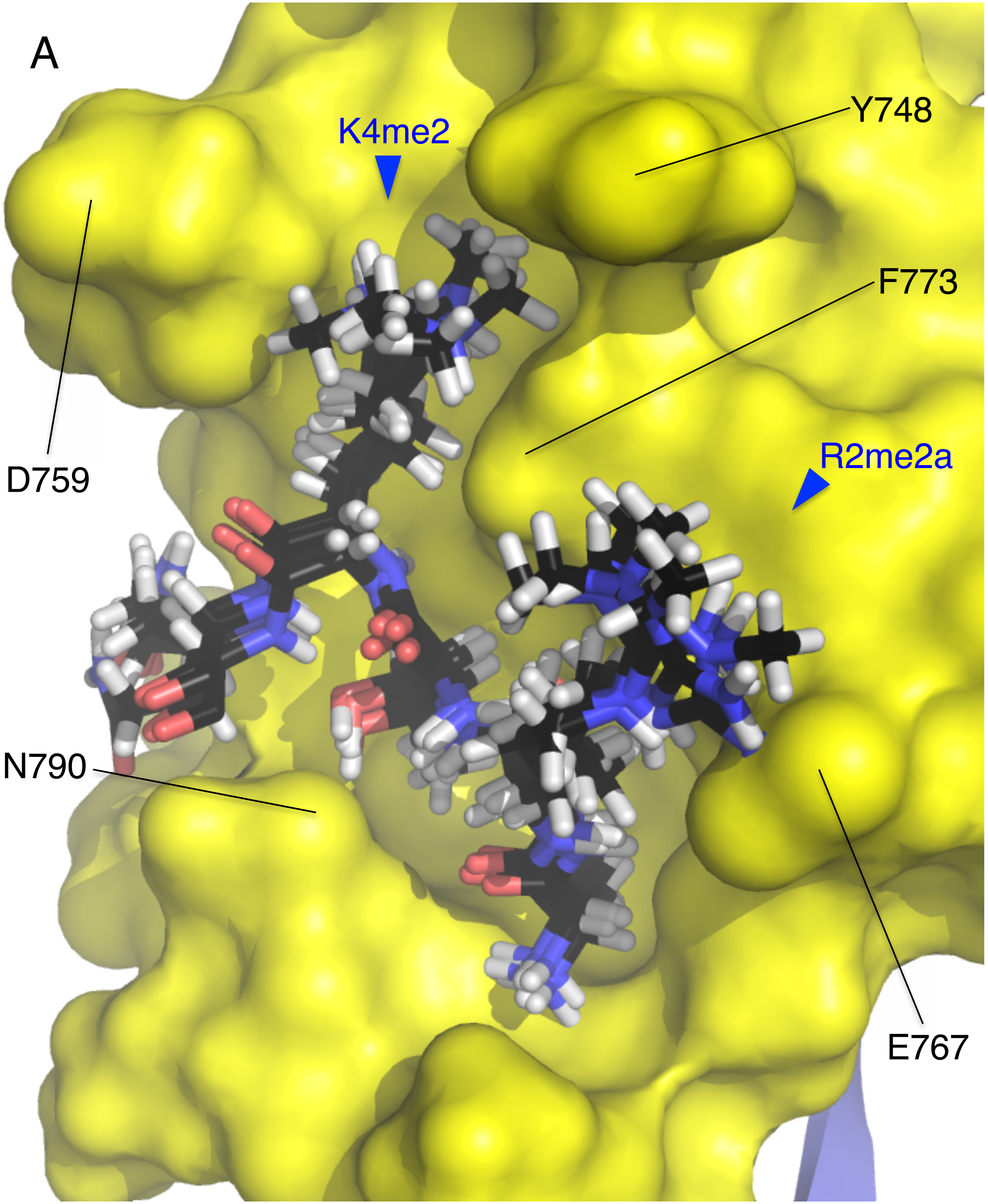

B

Buried surface area (Å^2)	1076.4 ± 40.0
Total interaction energy (kcal/mol)	-254.7 ± 27.7
van der Waals interaction energy (kcal/mol)	-43.7 ± 3.4
electrostatic interaction energy (kcal/mol)	-211.0 ± 27.5
desolvation energy (kcal/mol)	4.0 ± 1.4
backbone RMSD from reference structure (Å)	0.87 ± 0.27
ligand backbone RMSD from mean	0.71 ± 0.25

Figure S7

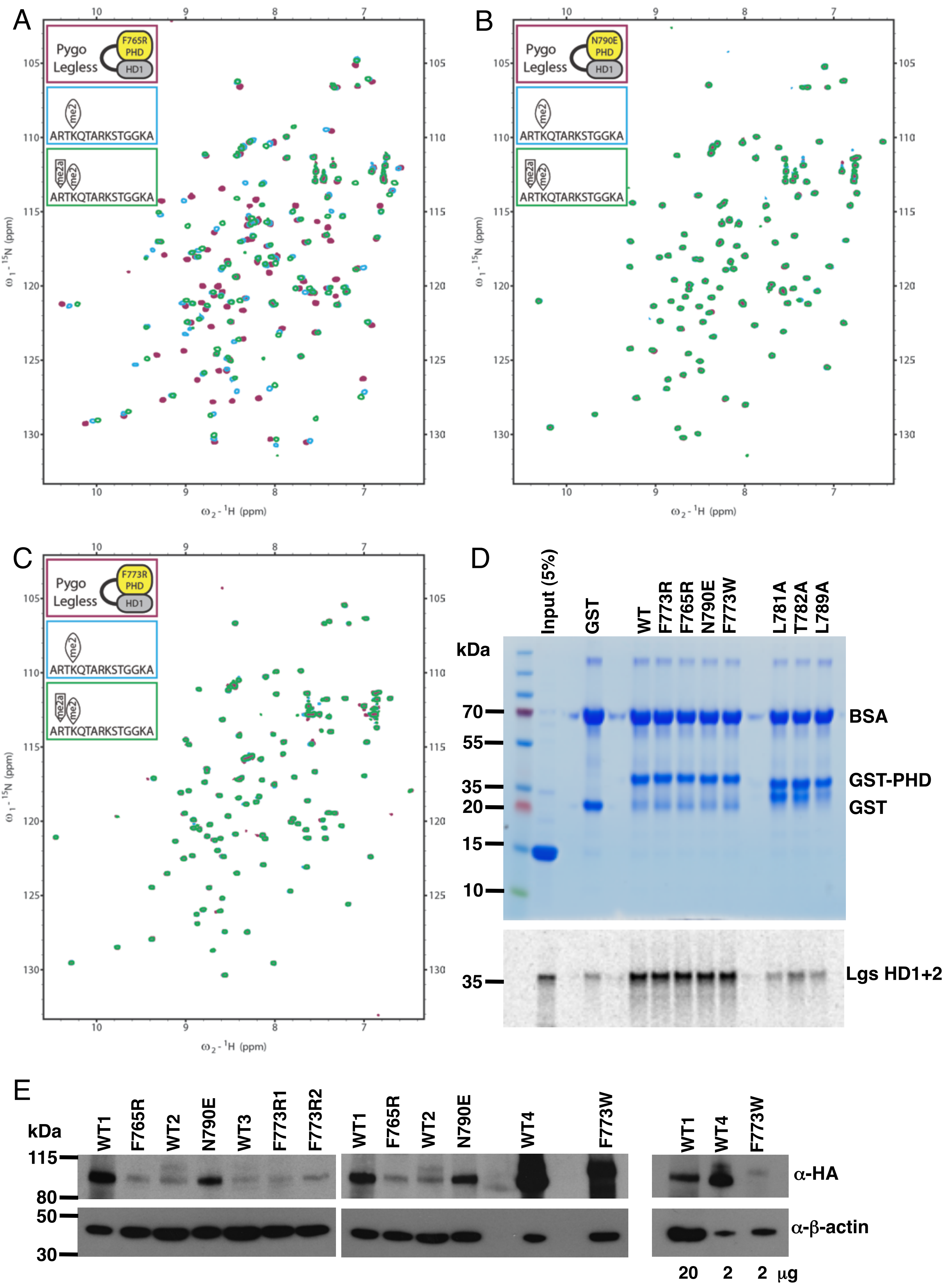
A
B
C
D
E
Pygo
Legless
F765R
PHD
HD1
N790E
F773R
ARTKQTARKSTGGKA
me2
me2a
ω1 - 15N (ppm)
ω2 - 1H (ppm)
Input (5%)
GST
WT
F773R
F765R
N790E
F773W
L781A
T782A
L789A
kDa
70
55
35
20
15
10
BSA
GST-PHD
GST
Lgs HD1+2
WT1
WT2
WT3
F773R1
F773R2
WT4
115
80
50
30
α-HA
α-β-actin
20
2
2 μg

Figure S8

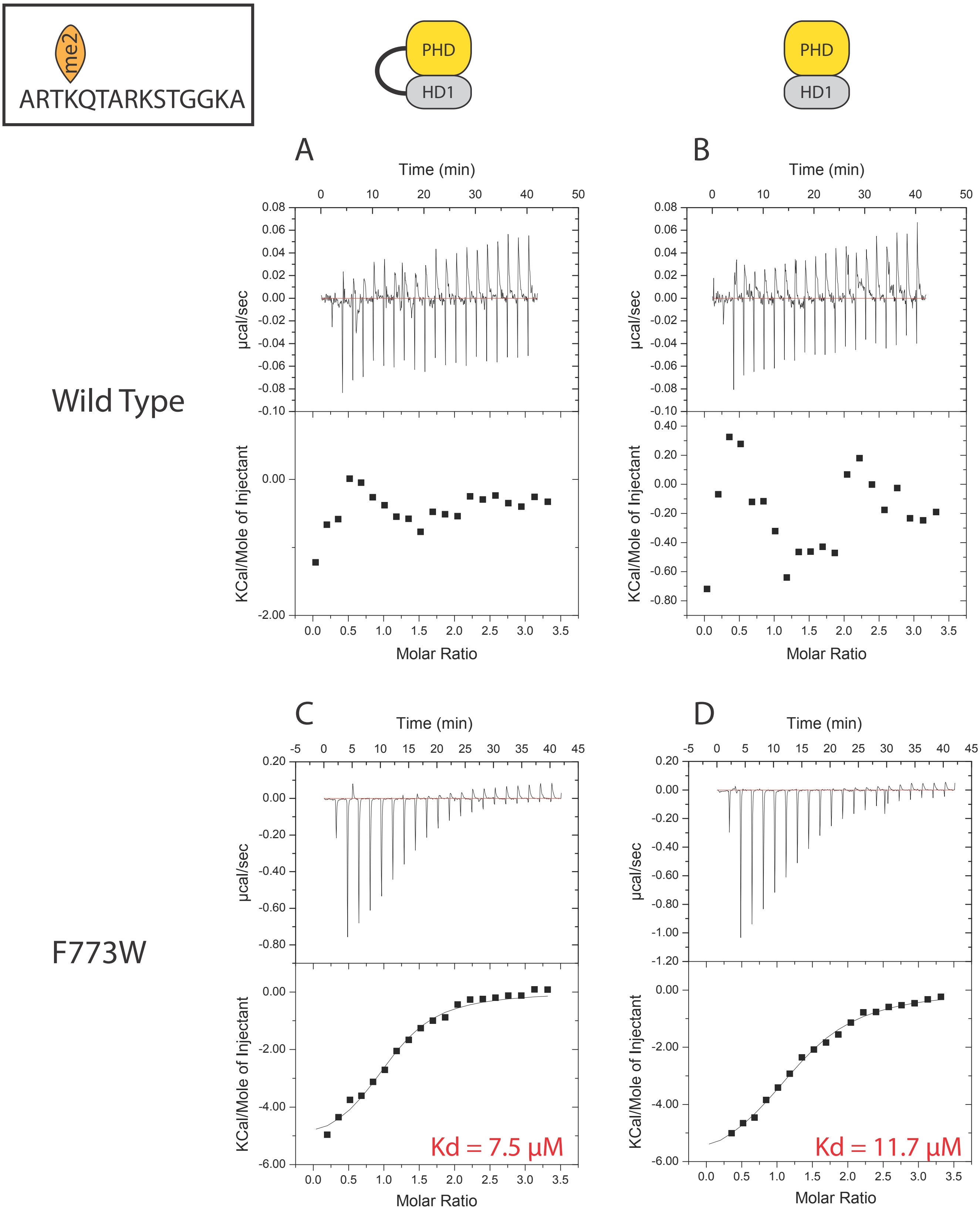

me2
ARTKQTARKSTGGKA
PHD
HD1
PHD
HD1
Wild Type
F773W
A
B
C
D
Time (min)
µcal/sec
KCal/Mole of Injectant
Molar Ratio
Kd = 7.5 µM
Kd = 11.7 µM

Figure S9

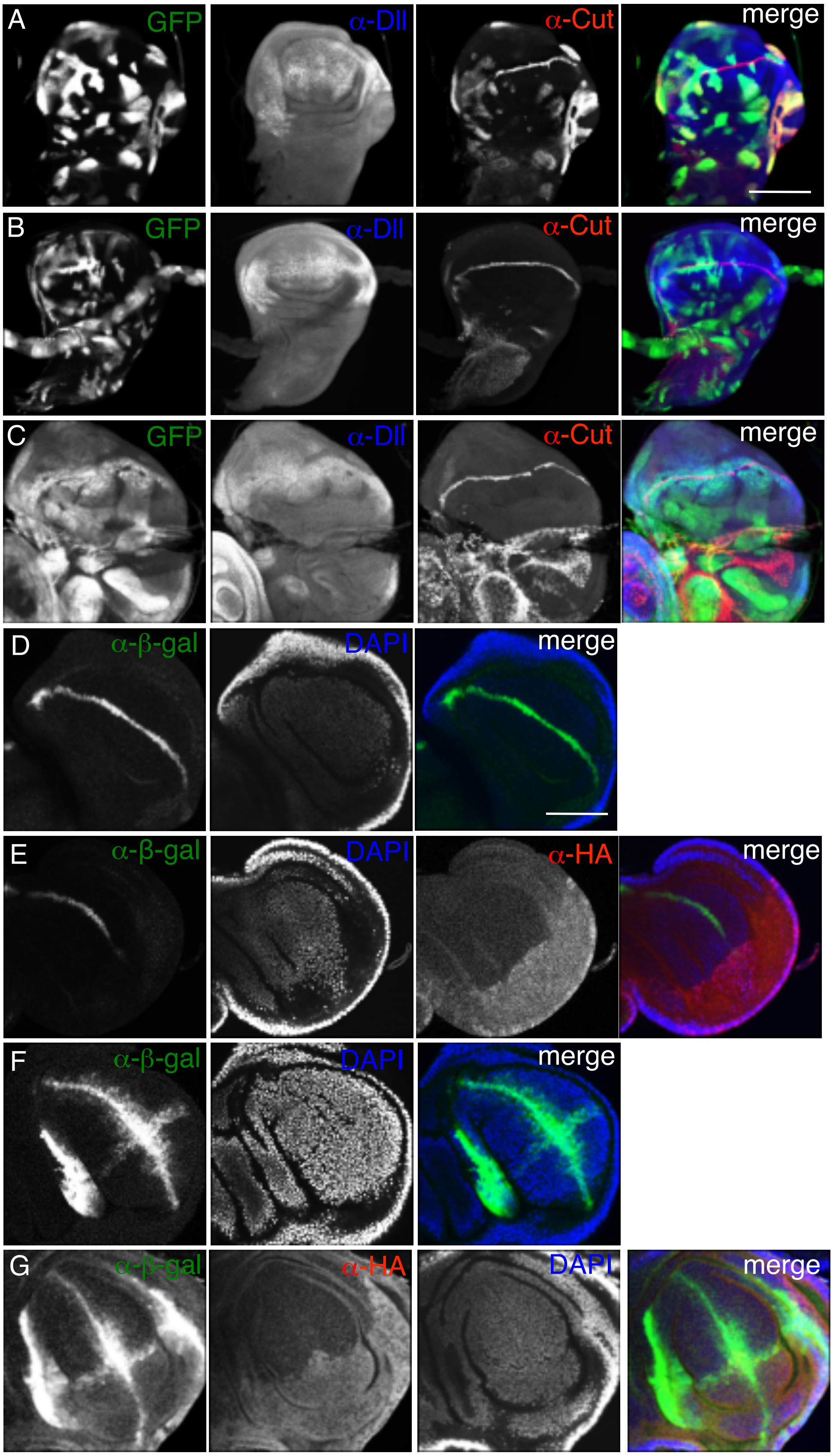
A
GFP
α-Dll
α-Cut
merge
B
GFP
α-Dll
α-Cut
merge
C
GFP
α-Dll
α-Cut
merge
D
α-β-gal
DAPI
merge
E
α-β-gal
DAPI
α-HA
merge
F
α-β-gal
DAPI
merge
G
α-β-gal
α-HA
DAPI
merge

Figure S10

SUPPLEMENTAL FIGURE LEGENDS

Fig. S1 (related to Fig. 1)

Conservation of Pygo PHD fingers amongst animal phyla. Alignments of PHD sequences of *bona fide* Pygo orthologs from 9 different phyla (of 35 known animal phyla), which contain a match to the Pygo signature motif EVND (magenta) and an NPF motif in their N-terminus (Thompson et al., 2002), except for those marked by @ for which the only known sequence is for the C-terminal PHD-containing fragment; green, pocket divider; blue, R2 binding site; grey, T3 channel; yellow, allosteric triplet (including PHD signature residue W); red, Zn^{2+}-coordinating residues; numbers indicate total Pygo residues (right), and first PHD residue (left). The following accession numbers were used: Amphimedon XP_003385401, Tribolium EFA11073.1, Trichoplax XP_002110816.1, Campotonus EFN72586, Ixodes XP_002413030.1, Hydra XP_002160262.1, Strongylocentrotus XP_791313.1, Danaus EHJ76837.1, Mnemiopsis ADO34165.1, Metuseiulus XP_003741865.1, Anopheles XP_003436178.1, Acyrthosiphon XP_001946189.2, Ciona XP_002128934.1, Trichinella XP_003381121.1, Nematostella XP_001629729.1, Solenopsis EFZ11656.1, Danio NP_001028283.2 (Pygo2), Xenopus AAM94597.1 (Pygo2), hPygo1 AF457207, hPygo2 AAH06132; Drosophila AAF57161, Ceratitis (medfly) XP_004529202.1; Trichuris (Tmu-pygo-1), Prionchulus (Ppu-pygo-1) (J. Pettitt, personal communication).

Fig. S2 (related to Table 1)

Comparison between *Drosophila* and human PHD-HD1 complex. Superimposition of backbones of (**A**) 18 dPHD-HD1 complexes found in the asymmetric unit and (**B**) *Drosophila* (PHD, blue; HD1,

yellow) and human PHD-HD1 complex (2vpb; hPygo1 PHD, magenta; BCL9 HD1, green). X-ray diffraction data were processed with Mosflm (Leslie, 2006) and scaled with Scala (Evans, 2006) (**Table 1**), and the *Drosophila* structure was solved by molecular replacement with Phaser (McCoy et al., 2005) based on 2vpb, and refined at 2.68 Å with Refmac (Murshudov et al., 1997). The models were updated with Coot (Emsley and Cowtan, 2004), and their stereochemistry was verified with MolProbity (Chen et al., 2010), and analyzed with the CCP4i programs (Winn et al., 2011). Note that the 18 *Drosophila* complexes in the asymmetric unit are structurally the same, and their consensus backbone is very similar to that of the human complex (rmsd value of 0.85 Å), with some differences due to different crystal contacts with symmetry-related molecules (mainly in the unstructured N-terminus of HD1, but also in its C-terminus).

Fig. S3 (related to Fig. 4)

NMR titrations of PHD-HD1link with histone H3 peptides. (A, B) Overlay of HSQC spectra of 50 μM ^{15}N-labelled PHD-HD1link incubated with increasing concentrations of (**A**) H3K4me2 or (**B**) H3R2me2aK4me2; 19 PHD residues with shift perturbation values of >0.1 at the top concentration are labelled; red, no peptide; orange, 120 μM; yellow, 250 μM; cyan, 600 μM; green, 1.2 mM; blue, 3.0 mM; spectra shown in main **Fig. 4** correspond to red (no peptide) and cyan (1.2 mM peptide). **(C)** K_d values (in μM) and standard errors (std err), derived from spectra shown in (**A, B**). K_d values were obtained by fitting the chemical shift perturbations for 5 different ligand concentrations (after minor adjustments of concentrations following amino acid analysis of peptides) to a quadratic equation for single-site binding, d_{obs} = 0.5*m1*(L_T+P_T+K_d-sqrt((L_T+P_T_K_d)^2-4*L_T*P_T)), using Kaleidagraph version 4.1, where L_T and P_T are the total concentrations of ligand and protein, and K_d and m1 are floating variables in the fit; median K_d values for the two peptides were derived from residues with

low standard errors (bold, std err <150). Note that the residues supporting the R2 groove floor (S768-C770) show considerably higher affinities to dually- compared to singly-modified peptide (median values 1.30 versus 3.29 mM, with the caveat that the values for the singly-modified peptide show relatively large fitting errors, an indication that this ligand does not interact properly with its cognate PHD surface), which might imply a preference of the R2 groove to accommodate H3R2me2aK4me2 over H3K4me2. **(D)** Kaleidagraph plots of selected residues for singly- (cyan, K4) or dually-modified (green, R2K4) peptide, as indicated in box (after fitting either to the change in ^{1}H or ^{15}N chemical shift for a given correlation peak, depending on which varied the greatest); percentage of bound protein (after normalization) is plotted against increasing peptide concentrations.

Fig. S4 (related to Fig. 4)

Lack of binding between PHD-HD1link and histone H3 peptides without methylated K4. Overlays of HSQC spectra of 50 μM ^{15}N-labelled PHD-HD1link + 1 mM H3R2me2a or unmodified histone H3 peptide (red) onto PHD-HD1link alone (black), as indicated above panels.

Fig. S5 (related to Fig. 5)

NOESY spectra from PHD-HD1 probed with histone H3 peptides. (**A**) Double half-filtered 2D ^{1}H-^{1}H NOESY spectrum from ^{13}C-^{15}N-labelled dPHD-HD1link (500 μM) probed with 3 mM unlabelled H3R2me2aK4me2, annotated with assignments to specific H-H contacts (see also **Fig. S6**). Note that the multiplicity of the observed NOE correlation peaks was independent of whether ^{15}N-decoupling pulses were applied in t_1, indicating that all NOE transfer originates from ^{1}H(^{12}C), and not ^{1}H(^{15}N). Furthermore, the traces above the panel, taken at the f_1 frequency indicated by the red

line, show that NOE cross-peak intensity was dependent upon NOE mixing time (τ_m), verifying that the peaks are derived from NOE transfer rather than from other artifactual transfer pathways. (**B**) Double half-filtered 2D ^{1}H-^{1}H NOESY spectra for H3R2me2aK4me2 (same spectrum as in **A**) or H3K4me2 (*second panel*), compared to control spectra derived from protein-only (500 μM dPHD-HD1 in deuterated aqueous phosphate buffer; *third panel*) or peptide-only (3 mM H3R2me2aK4me2 in deuterated aqueous phosphate buffer; *fourth panel*); boxed regions reveal crucial NOEs from histone peptide *N*-methyl groups and aromatic protons of dPHD-HD1link that are absent in the two control spectra (see also main **Fig. 5**).

Fig. S6 (related to Fig. 5)

Compilation of restraints derived from NOESY spectra and CSPs. Top, unambiguous restraints with regard to dually-methylated histone H3 peptide (A1 - Q5), derived from H-H intermolecular NOEs. Bottom, list of ambiguous restraints, derived from CSPs. Note that, due to low signal:noise in (HB)CB(CGCD)HD assignment spectra (Yamazaki et al., 1993) which correlate Cβ with aromatic proton resonances in the same residue, no signals were evident for F765, precluding an unambiguous assignment for its side chain. However, 4 unassigned cross-peaks from the *N*-methyl groups to aromatic protons are consistent with contacts involving the F765 phenyl ring (see **Fig. S5**), but since this assignment could not be confirmed independently, these 4 contacts were not included in the NOE restraints file used to guide the docking simulations.

Fig. S7 (related to Fig. 5)

Ensemble of HADDOCK models and refinement statistics. (**A**) Top 4 poses of single cluster, based on HADDOCK score, of dually-modified histone H3 peptide (in stick representation; amino acids 6 and 7 of histone H3 were omitted, for clarity; colors are as in main **Fig. 2**) bound to PHD-HD1link (surface representation, yellow), with key residues labelled. (B) Refinement statistics for 200/200 HADDOCK models in the single cluster.

Fig. S8 (related to Fig. 7)

Characterization of wt and mutant Pygo. (A-C) Overlay of HSQC spectra of 50 μM ^{15}N-labelled mutant PHD-HD1link (as indicated in panels) + 1 mM H3K4me2 (cyan) or H3R2me2aK4me2 (green) onto PHD-HD1link alone (magenta), indicating reduced binding of F765R (**A**), but complete loss of histone binding of N790E (**B**) and F773R (**C**); note the well-dispersed spectra of these mutants, confirming that they retain normal folding. **(D)** Top, SDS-PAGE of purified wt or mutant PHD-HD1link, as indicated above panel, used for pull-down assays with ^{35}S-methionine-labelled Lgs(HD1+2), as previously described (Townsley et al., 2004); the band at 15 kD in the input lane corresponds to globin (in the reticulocyte lysate used for *in vitro* labelling); 1 mg/ml bovine serum albumin (BSA) was added, to minimize unspecific binding. Underneath, autoradiogram of SDS-PAGE, revealing normal (wt) levels of Lgs binding of the histone-surface mutants used in this study; by contrast, Lgs binding mutants (L781A, T782A, L789A) show much reduced binding, as reported (Townsley et al., 2004), at the level of unspecific binding observed in the GST control lane. (**E**) Western blots of total embryonic extracts, prepared as described (Mendoza-Topaz et al., 2011), probed with antibodies as indicated on the right, to reveal expression levels of wt and mutant HA-

Pygo used in this study (F773W, Pygo-gof; positions of molecular weight markers on the left); 20 μg of total protein was used per lane, unless otherwise specified.

Fig. S9 (related to Fig. 8)

Binding of wt and gof mutant *Drosophila* PHD-HD1 to histone H3 peptide. ITC profiles for the binding of H3K4me2 (15-mer) to wt or F773W (gof) mutant PHD-HD1 complexes (amino acids 744-803 and 321-352, respectively), cloned in pETM30 as tandem domains separated by SGSLEVLFQGPGSG (containing a PreScission cleavage site), and purified with or without cleavage (as indicated above panels) after removal of the GST tag, and dialysis into 100 mM NaCl, 25 mM Tris-HCl pH 8.0, as described (Miller et al., 2010). Data were fitted to a one-site model, as previously described (Miller et al., 2010), and K_d values are given in the individual panels. Neither wt complex yielded reliable recordings, in contrast to a previously reported recording for the unlinked *Drosophila* complex obtained with a different ITC set-up, elsewhere (Fiedler et al., 2008) which also yielded 3-4x higher values for hPHD-HD1 (Miller et al., 2010). Note also that the linked PHD-HD1-gof complex exhibits a slightly higher affinity for H3K4me2 than the unlinked PHD-HD1-gof complex (which shows a marked tendency to dissociate), likely because of its 1:1 stoichiometry, demonstrating full functionality of the linked complex.

Fig. S10 (related to Fig. 8)

Pygo-gof acts through Notch targets. (**A-C**) Single confocal sections through wing discs as in **Fig. 8A, B**, bearing 'flip-on' clones (marked by GFP, green) that overexpress (**A**) Pygo-gof, (**B**) WT1 or (**C**) Arm-S10, triple-stained with antibodies against Dll (blue), Cut (red), and DAPI (to monitor the focal plane); merges

on the right. Note that the staining patterns with WT1 (**B**) are indistinguishable from control discs (without Pygo overexpression) since the Pygo overexpression in this line is considerably lower than that of WT4 (see **Fig. S6B**) shown in main **Fig. 8B** (so does not repress normal *cut* expression). (**D-G**) Single confocal sections of prospective wing blade territories within the young wing discs shown in main **Fig. 8C-F**, triple-stained with antibodies against β-galactosidase (green), HA (red) and DAPI (blue); (**D, E**) *cut-lacZ*; (**F, G**) *wg-lacZ*. Size bars, (**A-B**) 50 μm or (**D-G**) 25 μm.

SUPPLEMENTAL REFERENCES

Chen, V.B., Arendall, W.B., 3rd, Headd, J.J., Keedy, D.A., Immormino, R.M., Kapral, G.J., Murray, L.W., Richardson, J.S., and Richardson, D.C. (2010). MolProbity: all-atom structure validation for macromolecular crystallography. Acta crystallogr D Biol crystallogr *66*, 12-21.

Emsley, P., and Cowtan, K. (2004). Coot: model-building tools for molecular graphics. Acta Crystallogr D Biol Crystallogr *60*, 2126-2132.

Evans, P. (2006). Scaling and assessment of data quality. Acta Crystallogr D Biol Crystallogr *62*, 72-82.

Fiedler, M., Sanchez-Barrena, M., Nekrasov, M., Mieszczanek, J., Rybin, V., Muller, J., Evans, P., and Bienz, M. (2008). Decoding of methylated histone H3 tail by the Pygo-BCL9 Wnt signaling complex. Mol Cell *30*, 507-518.

Leslie, A.G. (2006). The integration of macromolecular diffraction data. Acta Crystallogr D Biol Crystallogr *62*, 48-57.

McCoy, A.J., Grosse-Kunstleve, R.W., Storoni, L.C., and Read, R.J. (2005). Likelihood-enhanced fast translation functions. Acta Crystallogr D Biol Crystallogr *61*, 458-464.

Mendoza-Topaz, C., Mieszczanek, J., and Bienz, M. (2011). The Adenomatous polyposis coli tumour suppressor is essential for Axin complex assembly and function and opposes Axin's interaction with Dishevelled. Open Biol *1*, 110013.

Miller, T.C., Rutherford, T.J., Johnson, C.M., Fiedler, M., and Bienz, M. (2010). Allosteric remodelling of the histone H3 binding pocket in the Pygo2 PHD finger triggered by its binding to the B9L/BCL9 co-factor. J Mol Biol *401*, 969-984.

Murshudov, G.N., Vagin, A.A., and Dodson, E.J. (1997). Refinement of macromolecular structures by the maximum-likelihood method. Acta Crystallogr D Biol Crystallogr *53*, 240-255.

Otting, G., and Wüthrich, K. (1989). Extended heteronuclear editing of 2D ^{1}H NMR spectra of isotope-labeled proteins, using the X(ω_1, ω_2) double half filter. J Magn Reson *85*, 586-594.

Thompson, B., Townsley, F., Rosin-Arbesfeld, R., Musisi, H., and Bienz, M. (2002). A new nuclear component of the Wnt signalling pathway. Nat Cell Biol *4*, 367-373.

Townsley, F.M., Thompson, B., and Bienz, M. (2004). Pygopus residues required for its binding to Legless are critical for transcription and development. J Biol Chem *279*, 5177-5183.

Winn, M.D., Ballard, C.C., Cowtan, K.D., Dodson, E.J., Emsley, P., Evans, P.R., Keegan, R.M., Krissinel, E.B., Leslie, A.G., McCoy, A., *et al*. (2011). Overview of the CCP4 suite and current developments. Acta crystallogr D Biol crystallogr *67*, 235-242.

Yamazaki, T., Forman-Kay, J.D., and Kay, L.E. (1993). Two-dimensional NMR experiments for correlating ^{13}Cβ and ^{1}Hδ/ε chemical-shifts of aromatic residues in ^{13}C-labeled proteins via scalar couplings. J Am Chem Soc *115*, 11054-11055.
